# Physics-based simulations of aerial attacks by peregrine falcons reveal that stooping at high speed maximizes catch success against agile prey

**DOI:** 10.1371/journal.pcbi.1006044

**Published:** 2018-04-12

**Authors:** Robin Mills, Hanno Hildenbrandt, Graham K. Taylor, Charlotte K. Hemelrijk

**Affiliations:** 1 Groningen Institute for Evolutionary Life Sciences, University of Groningen, Groningen, Groningen, Netherlands; 2 Department of Zoology, University of Oxford, Oxford, Oxfordshire, United Kingdom; Northeastern University, UNITED STATES

## Abstract

The peregrine falcon *Falco peregrinus* is renowned for attacking its prey from high altitude in a fast controlled dive called a stoop. Many other raptors employ a similar mode of attack, but the functional benefits of stooping remain obscure. Here we investigate whether, when, and why stooping promotes catch success, using a three-dimensional, agent-based modeling approach to simulate attacks of falcons on aerial prey. We simulate avian flapping and gliding flight using an analytical quasi-steady model of the aerodynamic forces and moments, parametrized by empirical measurements of flight morphology. The model-birds’ flight control inputs are commanded by their guidance system, comprising a phenomenological model of its vision, guidance, and control. To intercept its prey, model-falcons use the same guidance law as missiles (pure proportional navigation); this assumption is corroborated by empirical data on peregrine falcons hunting lures. We parametrically vary the falcon’s starting position relative to its prey, together with the feedback gain of its guidance loop, under differing assumptions regarding its errors and delay in vision and control, and for three different patterns of prey motion. We find that, when the prey maneuvers erratically, high-altitude stoops increase catch success compared to low-altitude attacks, but only if the falcon’s guidance law is appropriately tuned, and only given a high degree of precision in vision and control. Remarkably, the optimal tuning of the guidance law in our simulations coincides closely with what has been observed empirically in peregrines. High-altitude stoops are shown to be beneficial because their high airspeed enables production of higher aerodynamic forces for maneuvering, and facilitates higher roll agility as the wings are tucked, each of which is essential to catching maneuvering prey at realistic response delays.

## Author summary

Peregrine falcons are famed for their high-speed, high-altitude stoops. Hunting prey at perhaps the highest speed of any animal places a stooping falcon under extraordinary physical, physiological, and cognitive demands, yet it remains unknown how this behavioural strategy promotes catch success. Because the behavioral aspects of stooping are intimately related to its biomechanical constraints, we address this question through an embodied cognition approach. We model the falcon’s cognition using guidance laws inspired by theory and experiment, and embody this in a physics-based simulation of predator and prey flight. Stooping maximizes catch success against agile prey by minimizing roll inertia and maximizing the aerodynamic forces available for maneuvering, but requires a tightly tuned guidance law, and exquisitely precise vision and control.

## Introduction

The stoop is a remarkable attack strategy used by peregrine falcons *Falco peregrinus*, and a range of other raptors [1–4]. It involves a steep, controlled dive in which the attacker strikes its prey at high-speed with a massive blow in mid-air [1]. The high momentum of the attacker places it at obvious risk of harm, especially when diving into flocks of birds [1] or when pulling out only meters from the ground [3]. Arguably, for the stoop to evolve as an habitual attack strategy, these risks must be outweighed by certain survival advantages, and stooping has therefore been proposed either to save energy [5], or to enhance catch success [6]. These hypothetical advantages remain unproven, however, because it is challenging to compare the success rates of different attack strategies empirically. Success rates are confounded by a variety of factors, including the experience [1, 5] and reproductive status [7] of the attacker, the season of the attack [8], and the species of prey [6]. Even the seriousness of the attacker’s behavior may be an important source of variation: falcons seem to not always focus on achieving a high success rate, and appear sometimes to be practising or playing with their prey [9]. Moreover, the outcome of the stoop is often difficult to observe due to its high speed [6].

There presumably exists a trade-off between different factors influencing catch success in a stoop. On the one hand, it has been proposed that the high speed of the attack provides an element of surprise, leaving little time for the prey to evade [5, 10]. On the other hand, it is possible that the high speed of the attack decreases the precision of interception [2], and makes it harder for the attacker to follow the prey if it turns sharply [11]. Such trade-offs are difficult to investigate empirically, and we therefore turn to modeling and simulation. Because physical and physiological constraints influence catch success, we use an embodied cognition approach [12]. We investigate the success of different attack strategies by incorporating in a physics-based simulation model the aerodynamics, flight mechanics, guidance, and control. Such detailed simulations have already proven useful in work on missile guidance: the increasing demand for better performing missiles forces the inclusion of the detailed dynamics of the missile and its target when comparing the effectiveness of different guidance systems [13]. The nonlinear nature of these dynamics restricts the use of analytic methods, such as linear-quadratic optimal control, and the effectiveness of different mechanisms must therefore be examined through parametric variation of the system between repeated simulations of interception. Here, we study the general intercept problem under the particular dynamics of flapping and gliding bird flight. Interception in this biological context differs from that of missiles in that gravity plays a pivotal role in determining the best attack strategy: in missiles, the speed and acceleration are so high that the effects of gravity are marginal, but in birds, the acceleration due to gravity dominates the dynamics.

**Fig 1:**
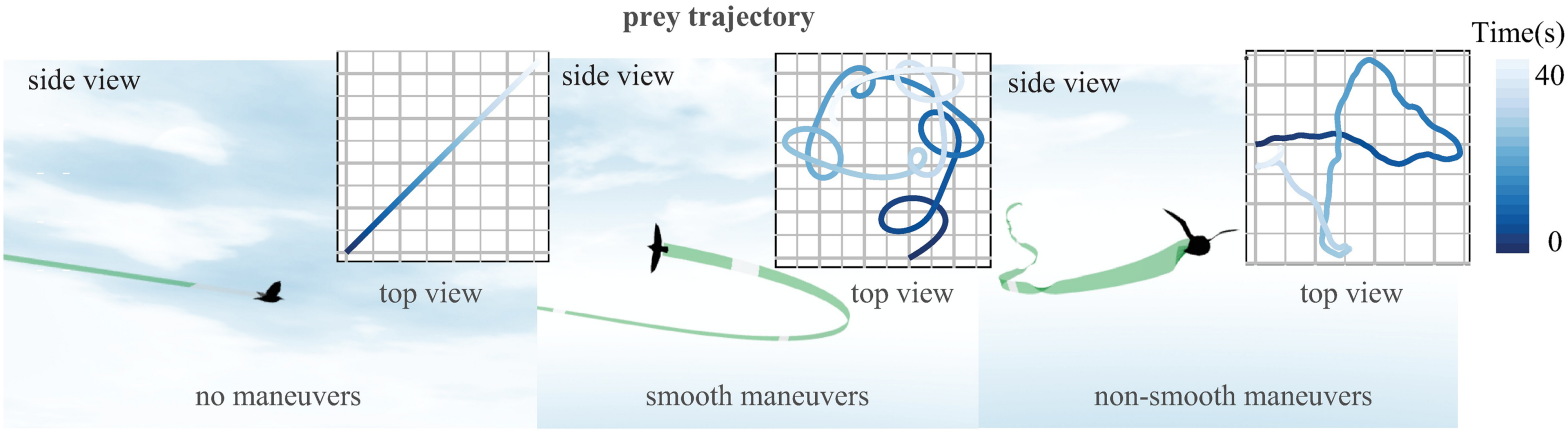
Examples of prey motion in the no maneuver, smooth maneuver and non-smooth maneuver condition.

The flight performance of the model-birds in our simulations depends on their flight morphology, and differs considerably between predator and prey. To maneuver, model-birds flap, glide, and vary their wing span. We use the model to study attacks by peregrine falcons on a habitual prey species, the common starling *Sturnus vulgaris*. We simulate three different patterns of flight by the prey—straight flight, smooth turning, and non-smooth turning (see Fig 1). This approach to varying the target motion is standard in simulations of missile guidance systems, and broadly summarizes the main options for the target to maneuver [13, 14]. It also captures the range of different prey behaviors found in nature. For instance, if a bird is caught by surprise while commuting, then typically it will be flying in a straight line. Conversely, when turning, birds usually maneuver smoothly, but they will also fly erratically if a threat is detected, in a kind of non-smooth maneuvering flight known as jinking. Here we investigate whether, when, and why stooping increases catch success in each of these three situations (see S1–S5 Videos for a visualization of attacks in each scenario).

**Fig 2:**
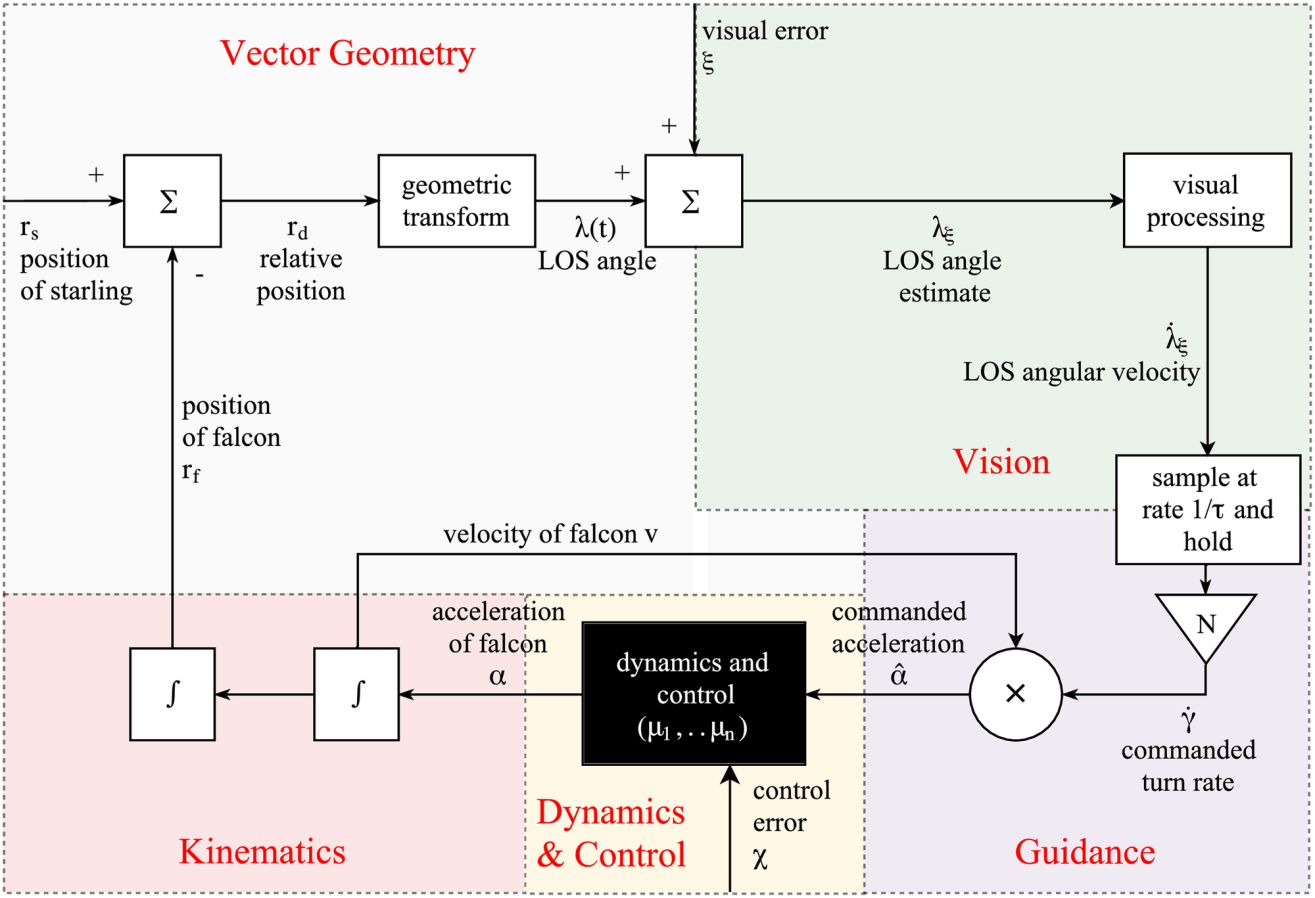
Block diagram of the feedback-loop in model-falcons. This diagram is intended to communicate the general structure of the model. A detailed explanation of the model equations is provided in Materials and methods. The boxes denote transfer functions, and additional parameters of the functions are noted in between brackets. Most of the feedback loop is generic, except for the detailed implementation of flapping flight contained in the black box labelled “dynamics and control”. A brief summary of the feedback loop now follows, in which we walk through each of the different segments of the feedback loop summarised as “Vision”, “Guidance”, “Dynamics and Control”, “Kinematics”, and “Vector Geometry”. Vision: to determine how it should turn, the falcon first extracts the line-of-sight angle λ, which is measured subject to visual error *ξ*. The measured line-of-sight angle λ_*ξ*_ is subsequently transformed into an angular velocity vector ${\dot{\lambda }}_{\xi }$ that denotes the estimated rate of change in the line-of-sight. The resulting signal from the visual system is fed to the guidance function every time interval *τ*, as denoted by the block labelled “sample … and hold”. Note that we also test an alternative implementation of visual processing delay in the model (continuous and delayed, instead of in discrete update intervals), as little is known about the nature of delay in birds. Results using either form of delay are highly similar (see S1 Fig). Guidance: the falcon’s guidance system multiplies the estimated line-of-sight rate ${\dot{\lambda }}_{\xi }$ by the navigation constant *N* to obtain the commanded change in the angle of the falcon’s velocity $\dot{\gamma }$ (see Eq 1), and the cross product is taken with the velocity of the falcon to obtain the commanded acceleration $\hat{\alpha }$. The dynamics and control function depends on the morphological parameters *μ*_1_, … *μ*_*n*_ and manipulates the wing shape and motion to produce an acceleration *α* which maximizes the forward acceleration whilst meeting the commanded acceleration as closely as possible (see Materials and methods section D.2 and E for detailed model equations). Kinematics and Vector Geometry: the acceleration of the falcon *α* is integrated in the kinematics section and fed back to the visual system through the medium of the vector geometry needed to relate the line-of-sight angle to the updated positions of the model-falcon and model-starling. Note that the segment of the block diagram labeled “Vector Geometry” operates outside of the model-falcon, so we do not imply that the falcon cognitively represents either its own position or that of its target. In particular, the falcon has no knowledge of—and no need to know—the distance to its target; all that the falcon needs to know is the direction of its target as measured visually by the line-of-sight angle, and its own velocity, which is needed to determine the commanded acceleration from the commanded turn rate. Model-starlings have a similar control-loop, in which the segments of the feedback loop labelled “Vision” and “Guidance” are replaced by a forcing function *ζ*(*t*) that determines their (desired) trajectory (see Materials and methods section C).

Because falcons often attack in wide, open spaces at high altitude, there are no objects or boundaries in our simulation space. We parametrically vary the falcon’s initial position relative to its prey to simulate a continuum of possible attack strategies (e.g. stoops versus level chases). Predator and prey are each free to move with 6 degrees of freedom in translation and rotation, and are subject to gravitational and aerodynamic forces, which they manipulate by controlling their wings (Fig 2). The model-bird’s flight controller determines the changes in wing shape and motion that best meet the accelerations commanded by its guidance system, under a quasi-steady blade element model of the aerodynamics. In model-falcons, the guidance system commands turning toward the prey in closed-loop, whereas in model-starlings the guidance system is a forcing function that is set to ensure that the prey remain within approximately ±20 m of their starting altitude. Because we assume that birds maximize their flight speed during escape and pursuit, model-birds always generate the maximum possible forward acceleration given their instantaneous velocity and orientation, subject to the constraint that they must simultaneously meet, as closely as possible, the acceleration demanded normal to their flight direction by their guidance system.

The falcon’s closed-loop guidance is essential in commanding the changes in velocity that are needed to intercept prey, whether maneuvering or not, and to deal with the effects of steering error. Our model-falcons use a guidance law called pure proportional navigation, which has been shown to fit the empirically measured attack trajectories of peregrine falcons closely [15]. Proportional navigation is also favored as a guidance law in missiles, because it provides a simple way of implementing the geometric rule known as parallel navigation or constant absolute target direction (CATD), according to which the attacker holds the geographic direction of the line-of-sight to target constant through time [14, 16–18]. This geometry guarantees interception if the attacker is closing range, because at every instant it is set on a collision course with its target (i.e. would hit its target if both continued flying at constant velocity thereafter). Under pure proportional navigation, the attacker turns at an angular rate proportional to the angular rate of the line-of-sight to target. Although this guidance law can be written in three-dimensional vector form, it is more intuitively explained in the two-dimensional case, for which:

$$\begin{array}{c} \dot{\gamma }=N\dot{\lambda } \end{array}$$ (1)

where *γ* denotes the bearing of the attacker’s velocity vector and λ denotes the bearing of the line-of-sight to target, both measured in an inertial reference frame; the dot notation denotes the time derivative, and *N* is called the navigation constant. The numerical value of *N* determines the rate of convergence to a parallel navigation (CATD) course: in missiles, low values of *N* result in slow convergence, whilst high values can cause overshoot, leading to control instability [14]. Partly for these reasons, intermediate values of *N* between 3 and 5 are typical in most missile applications.

The overall objective of our simulations is to identify the attack strategy that maximizes the catch success of the falcon for a given prey motion, a range of assumptions regarding the delay in the falcon’s response, and the error in its vision and control (see Materials and methods). Here, an attack strategy is defined as some particular combination of the predator’s navigation constant *N*, and its initial vertical and horizontal distance from the prey. The optimization was conducted via parametric variation of the attack strategy, in combination with Generalized Additive Modeling (GAM), which we used to interpolate between the 10^6^ randomly chosen attack strategies that we simulated in each optimization [19, 20]. We hypothesise that stooping maximizes catch success, and that it does so as a direct consequence of the flight physics in our simulation model. We test this by asking whether a model-falcon’s catch success is maximized by attacking from a high altitude, which couples into a high flight speed in our simulations. Remarkably, we find that the optimality of stooping depends not only on the motion of the prey, but also on the tuning of the underlying guidance law. Specifically, we show that stooping is only expected to evolve in conjunction with the same low values of the navigation constant *N* that have been identified empirically in peregrine falcons [15].

## Results

### Starlings outmaneuver falcons in slow flight

**Fig 3:**
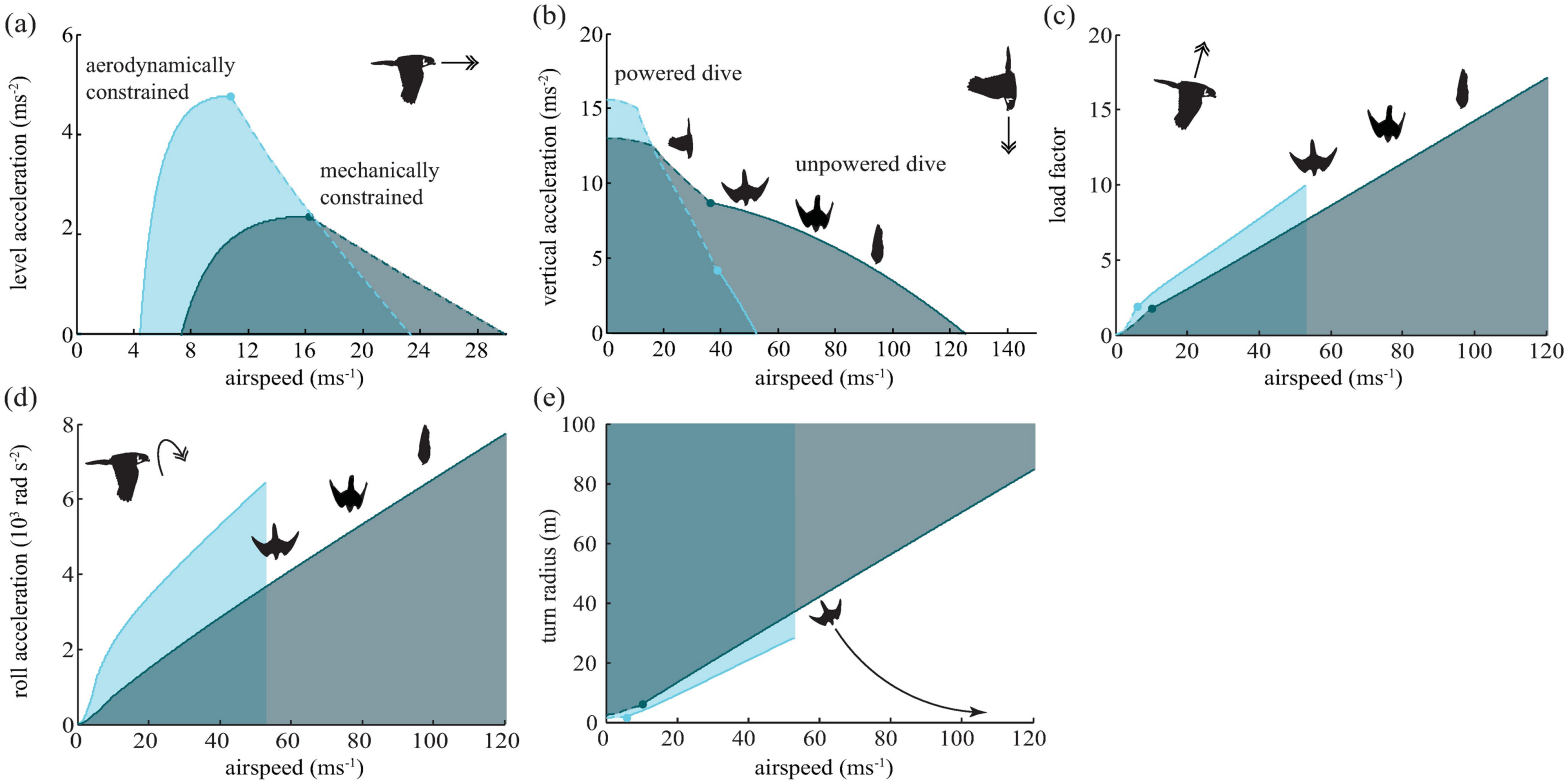
Flight performance graphs in the flight simulator for the peregrine falcon (dark blue) and the common starling (light blue). The double arrows denote the direction of acceleration displayed in the graph. The starling is able to outmaneuver the falcon at a given airspeed, if there exists a region under the curve of the starling that is not overlapping with that of the falcon. (a) Level acceleration versus air speed: level flight with the requirement that lift equals weight. Dashed lines denote the speed wherein torque forces constrain the maximum acceleration (mechanical constraints). Top level flight speed is reached at the point where level acceleration is zero. (b) Vertical dive acceleration (including gravity) versus air speed. At the end of the dashed lines, flapping is substituted by gliding with retracted wings in order to maximize vertical acceleration. (c) Load factor versus air speed. The load factor is defined as lift divided by weight. The maximum load factor does not scale quadratically with forward speed due to constraints in torque forces [11]. Instead, wings are retracted optimally to increase maximum load. (d) Roll acceleration versus air speed. Roll acceleration determines the speed with which the bird can redirect its lift and is calculated by estimating the whole-body inertia around the roll-axis and the maximum net torque production [11]. (e) Turning radius is calculated as the square of air speed divided by the maximum normal acceleration.

Flight performance is expected to be a key determinant of catch success in a chase. Clearly, any prey species that can fly faster than a falcon will be able to outrun its attacker in straight flight. In practice, peregrine falcons fly much faster than starlings, and our aerodynamic model predicts that they hold a considerable speed advantage in both level flight (maximum speed: 29 *versus* 23 ms^−1^; Fig 3a) and vertical dives (terminal speed: 123 *versus* 52 ms^−1^; Fig 3b; see section K for a comparison between flight performance in the model and empirical measurements). Even so, escape is possible if the prey species can outmaneuver its attacker. For instance, if at a given flight speed the prey can produce a higher aerodynamic force relative to body weight than its attacker (i.e. produce a higher load factor), then it may escape by turning more tightly than its attacker in a smooth maneuver called a turning gambit [21, 22]. Our aerodynamic model shows that starlings can indeed sustain higher load factors than peregrine falcons flying at the same speed (Fig 3c), and that although falcons can achieve even higher load factors by flying faster (Fig 3c), the net effect is such that a starling will always be able to turn on a tighter radius than a faster-flying falcon (Fig 3e). Similarly, if the prey can achieve a higher roll acceleration than the falcon, then it will be able to redirect its lift faster, and hence outmaneuver its attacker in a non-smooth jinking maneuver. Our aerodynamic model predicts that a starling can indeed produce a higher roll acceleration than a falcon flying at the same speed (Fig 3d). So great is a model-starling’s advantage in this respect that a model-falcon can only be expected to match a model-starling’s maximum roll acceleration by diving at close to terminal velocity (i.e. at close to its maximum speed). Hence, model-starlings may often escape model-falcons in our simulations, even though their maneuvers are not implemented as an evasive response to the falcon. In summary, a starling can always outmaneuver a falcon that is flying at a similar speed, but a falcon can always beat the load factor and roll acceleration of a starling by diving at a sufficiently higher speed. Whether this strategy enhances catch success will presumably depend on the flight pattern of the prey, and the complex ways in which the predator’s flight speed, response delay, and errors in vision and control interact to affect its guidance. We explore the outcome of these complex interactions in the simulations presented below.

### Stooping maximizes catch success if the prey maneuvers

**Fig 4:**
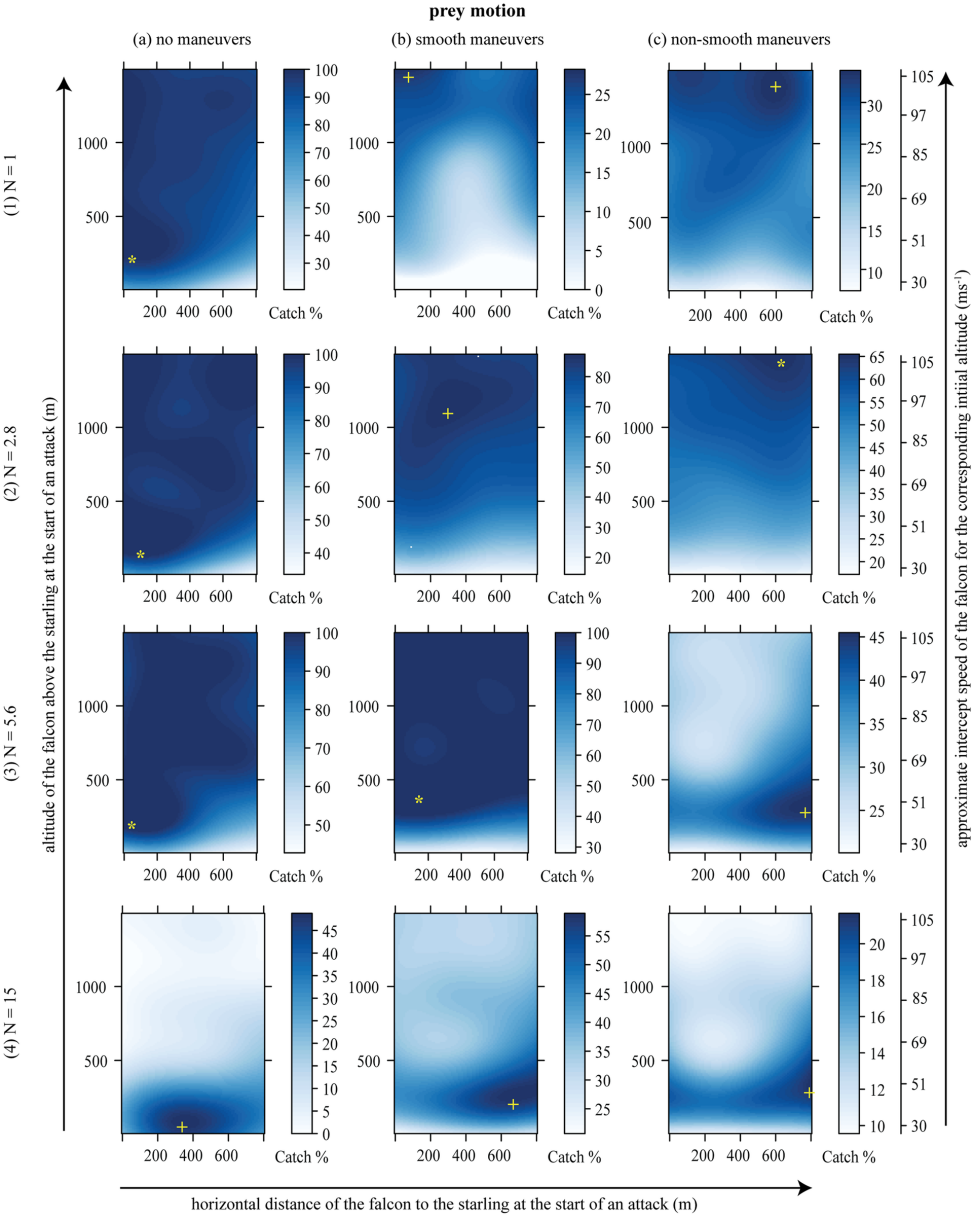
Catch success mapped onto initial altitude and horizontal distance from the prey for non-maneuvering, smooth maneuvering and non-smooth maneuvering prey, and for 4 values of the navigation constant *N*: A low extreme (*N* = 1), the optimal value for catching non-smooth maneuvering prey (*N* = 2.8), the optimal value for catching smooth maneuvering prey (*N* = 5.6), and a high extreme (*N* = 15). The yellow asterisks depict the global optima with respect to attack position and *N*, showing the attack strategy which uniquely maximizes catch success for a given prey motion. The yellow crosses denote local optima for a given *N* and prey motion. The approximate intercept speed corresponding to the initial altitude is shown on the right of the graph. This is only an approximate relationship because the exact intercept speed depends on many factors within each hunt.

The catch success of model-falcons was always maximized by entering a steep dive, but the optimal starting altitude varied greatly between the three different flight patterns of the prey (Table 1; see asterisked points in Fig 4). Catch success in attacks on straight-flying prey (Fig 4a) was maximized by stooping from a low altitude (< 200m), leading to a low flight speed at the point of intercept (35 − 45ms^−1^). Optimal stoop altitude was somewhat higher (*c*. 350m) when prey maneuvered smoothly (Fig 4b), leading to a moderate intercept speed (50 − 55ms^−1^). Catch success with non-smoothly maneuvering prey (Fig 4c) was maximized by stooping from a very high altitude (*c*. 1500m), leading to a very high intercept speed (> 100ms^−1^) approaching the terminal velocity of the model-falcon (see Fig 3). Interestingly, catch success barely declined when the model-falcon attacked from a higher altitude than the optimum (Fig 4), but was greatly reduced if the model-falcon attacked from a lower starting position (Fig 4), so stooping from a high altitude is never a bad strategy provided that the guidance system is appropriately tuned (see below).

**Table 1:** Attack strategies with maximum catch success.

prey motion	navigation constant *N*	altitude (m)	horizontal distance (m)	speed at interception (ms^−1^)
no maneuvers	1–6	150–200	70–90	35–45 (Low-speed)
smooth maneuvers	5.6	350	0–200	50–55 (moderate-speed)
non-smooth maneuvers	2.8	1500	641	>105 (high-speed)

### Optimization of the navigation constant for stooping

**Fig 5:**
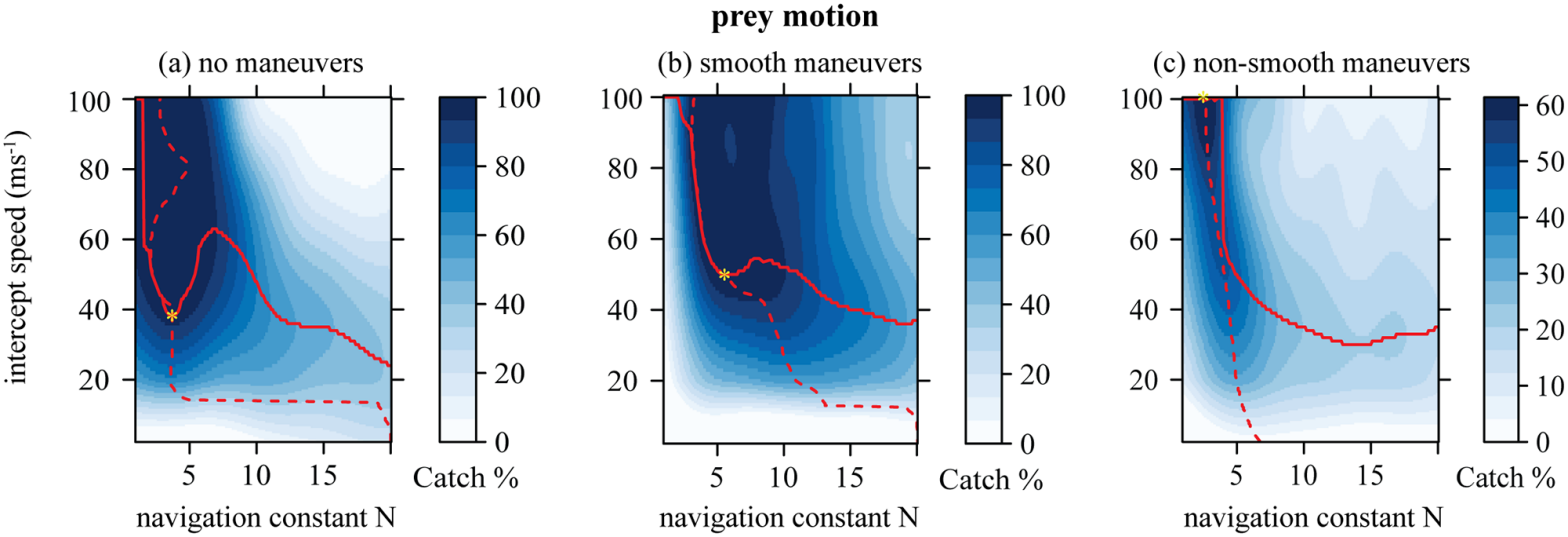
Catch success mapped onto intercept speed and navigation constant *N* for (a) non maneuvering, (b) smooth maneuvering and (c) non-smooth maneuvering prey. The solid line denotes the optimal interception speed for a given *N* and the dashed line denotes the optimal *N* for a given interception speed. The asterisks denote the global optimum with respect to intercept speed and *N* for a given prey motion.

The attack strategy of a model-falcon encompasses both its initial position relative to the prey, and the setting of its navigation constant *N*. The global optima that we have so far discussed (asterisked points in Fig 4) assume joint optimization of the predator’s initial attack position and its navigation constant *N*, and the optima for both parameters depend on the motion of the prey (see also Table 1). Selection on *N* is expected to be strongest when prey execute non-smooth maneuvers, for which high catch success is achieved over only a narrow range of *N* (compare width of dark blue area denoting high catch success in Fig 5c with the equivalent areas in Fig 5a and 5b). Interestingly, for all three types of prey motion, the optimal setting of *N* tends to be lower the faster the stoop (see dashed lines in Fig 5 plotting the optimal setting of *N* conditional upon the speed at intercept). Conversely, for a given setting of *N*, the optimal intercept speed becomes lower the higher the value of *N* (see solid lines in Fig 5 plotting the optimal speed at intercept conditional upon the setting of *N*). Thus, for any given type of prey motion, high-speed, high-altitude stoops *only* maximize catch success over a small range of comparatively low values of *N*. At higher values of *N*, catch success is maximized by using a low-speed (Fig 5), low-altitude (Fig 4) attack, but this is generally less successful than a high-speed, high-altitude attack at a lower value of *N*.

In summary, it turns out to be essential for our model-falcons to set their navigation constant appropriately: if a sub-optimal value of *N* were used, then stooping might no longer be the best attack strategy, because of poor catch success. For instance, if a model-falcon were to use the optimal value of *N* for smoothly maneuvering prey (*N* = 5.6) against prey executing non-smooth maneuvers, then a high-altitude stoop would be unlikely to result in prey capture (third panel of Fig 4c). This does not necessarily mean that a falcon must actively adjust *N* to match the maneuverability of its prey: the best attack strategy of a model-falcon against the best defensive flight pattern of a model-starling (i.e. non-smooth) involves entering a high-speed, high-altitude stoop at *N* ≈ 3. This minimax strategy not only yields maximal catch success against non-smoothly maneuvering prey, but also yields near-maximal catch success against prey that are flying straight or maneuvering smoothly (second row of Fig 4). Hence, subject to the assumptions of our model, we expect falcons to adopt a general strategy of stooping from high-altitude at *N* ≈ 3, because this strategy is effective against all of the different patterns of prey flight that we have tested here.

Some interesting flight trajectories emerge at *N* < 2 (see S2 Fig). In this case, the model-falcon exerts most of its acceleration towards the end of its attack (see also [14]), often diving below its prey before looping upward to intercept. This upward-curved trajectory is regularly observed in nature [4, 23], and has previously been suggested to be a strategy of a falcon to fly into the blind spot of its prey’s vision [9]. Our model provides a more parsimonious explanation for these flight paths, which can emerge naturally from the dynamics of the underlying feedback law.

### Response delays and errors in vision and control drive the need to stoop

**Fig 6:**
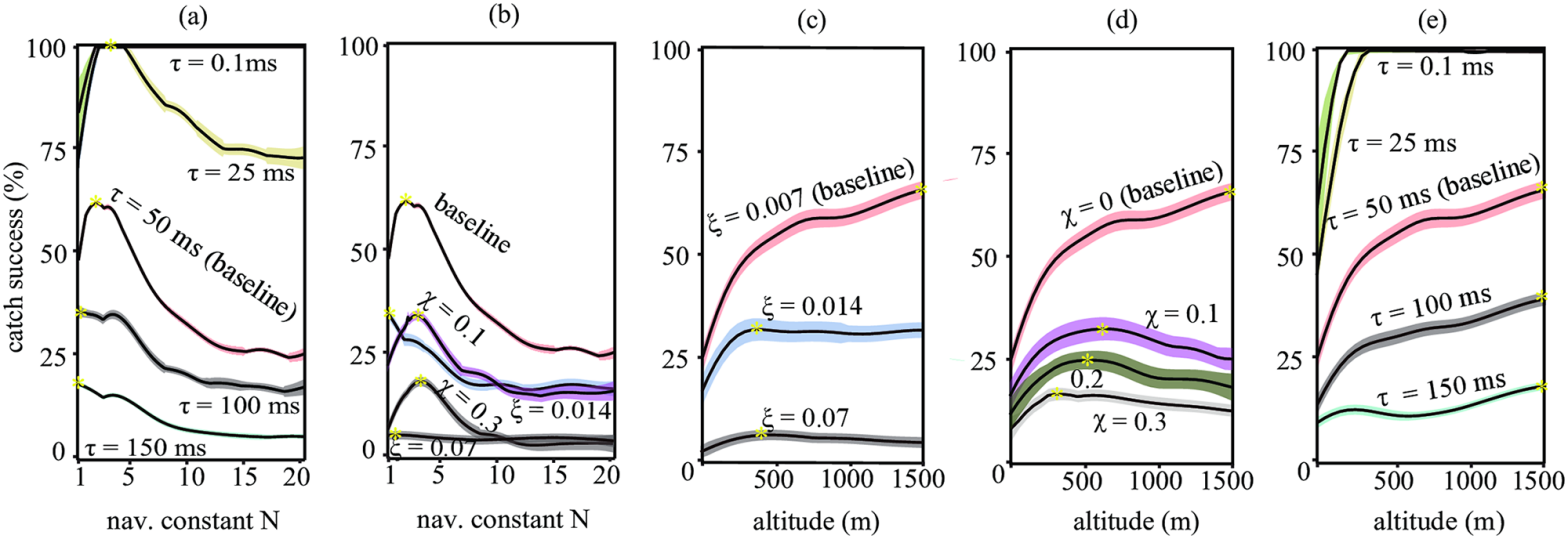
Relationship between catch success and various model parameters. Graphs depict results for non-smooth maneuvering prey, because in this condition the high-speed stoop with a low *N* shows the most marked increase in catch success for the falcon. The upper bounds in values for reaction times and errors in vision and control are chosen such that they are different enough to show substantial variation in simulation results, but remain low enough to allow for capture. (a) Maximum catch success as a function of *N* in the baseline model, for smaller (*τ* = 0.1*ms* & 25*ms*) or larger (*τ* = 100*ms* & 150*ms*) response delays, assuming the optimal attack position. The margins depict the 95% confidence intervals of the GAM. The asterisks denote the global optimum with respect to the x-axis. (b) Maximum catch success as a function of *N* for different visual (*ξ*) and control error (*χ*). (c) Maximum catch success as a function of altitude, for the baseline and for increased error in vision. (d) Maximum catch success as a function of altitude for various values of control error. (e) Maximum catch success as a function of altitude, for various values of response delay *τ*.

The most important factor that causes the reduction in catch success observed at high values of the navigation constant *N* is the response delay of the model-falcon. A robustness analysis (Fig 6a) shows that high values of *N* are no longer associated with a low catch success if the reactions of the falcon are effectively instantaneous (compare catch success as a function of *N* at *τ* = 0.1ms delay with the equivalent line for the default *τ* = 50ms delay used in our baseline model; see Materials and methods). Conversely, if the falcon’s actual response delay is greater than the default assumed in our baseline model, then the optimal value of *N* is driven towards an even lower value (Fig 6a). Visual error also affects the optimal value of *N* in our simulations: if the falcon is subject to greater visual error than the default value assumed in our model, then the navigation constant is again driven towards an even lower value of *N* (Fig 6b). This reflects the fact that the propagation of this visual error into the commanded acceleration is directly proportional to *N* (see Fig 2). In contrast, the optimal value of *N* is robust to the error assumed in the control system itself (Fig 6b).

How do response delays, or errors in vision and control, impact the success of a given attack strategy? As expected, catch success declines as each of these quantities increases (Fig 2). Remarkably, however, the optimal starting altitude becomes lower when the visual error or control error is increased (Fig 6c and 6d). Thus, a high-speed, high-altitude stoop only maximizes catch success if the falcon is accurate in both vision and control. A high-speed stoop maximizes catch success for all of the response delays that we tested, noting that any much longer delay would have resulted in very low catch success (Fig 6e). On the other hand, if the falcon’s response is effectively instantaneous (*τ* = 0.1ms), then 100% catch success is attained even in a low-altitude dive from < 200m. This implies that the falcon’s flight performance is sufficient to catch a starling in a low-level stoop, but that delays in the model-falcon’s response hamper its ability to catch prey. The lower catch success that results from having a slower response can be ameliorated by diving from higher altitudes at lower *N*.

### What mechanisms underlie the increased catch success in a stoop?

When a falcon stoops from high altitude, its attack is characterized by both a very high flight speed, and a very steep descent angle—either of which could promote catch success. To investigate the effect of steepness of the descent, we altered the initial conditions of the simulations so as to model a horizontal attack at very high initial speed (112 ms^−1^). This effectively simulates the final approach of a falcon that stoops from a very high altitude to gain speed before levelling off to intercept. Remarkably, the maximum catch success of these model-falcons is only 3% lower than for those intercepting their prey at the same speed in a steep dive (61 vs 64%). This implies that the steep descent angle is not directly responsible for the overwhelming success of a stoop, and hence that the key reason for starting from a very high altitude is to gain airspeed by converting potential energy to kinetic energy.

The very high airspeed attained in a stoop enables model-falcons to exceed the model-starlings’ maximal load factor and roll acceleration (Fig 3). To test which of these two dimensions of flight performance causes an increase in catch success in a stoop, we artificially capped the maximal load factor or maximal roll acceleration of our model-falcons. We thus investigated the catch success of a bird flying at the same high speed achieved in a high-altitude stoop (> 100ms^−1^), but with the lower maximal acceleration associated with sustained level flight (30ms^−1^). Limiting either component of the model-falcon’s flight performance resulted in a substantial drop in catch success (51% when limiting roll acceleration and 42% when limiting load factor). This suggests that the high speed that a falcon attains in a stoop is important partly because of the higher load factors and higher roll accelerations that can be achieved in high-speed flight. Interestingly though, model-falcons flying at high speed still performed considerably better than model-falcons in sustained level flight (31% vs 26%) even though the maximal load factor and roll acceleration was made the same, and even when these fast falcons levelled off before interception. This implies that a high flight speed is beneficial in and of itself, independent of the higher acceleration performance that is usually also associated with fast flight.

This result might seem surprising, because model-falcons fly faster than model-starlings even in sustained level flight (Fig 3a), and the most obvious consequence of increasing the falcon’s flight speed further is to increase its turning radius, potentially causing it to overshoot when attacking sharply turning prey. However, the flight speed of a falcon varies continuously in our model, on account of its varying acceleration demand, and work on missile guidance and control has shown that the accelerations commanded in response to variations in speed are lower when the angle between the current line-of-sight and the expected point of intercept is smaller. The faster the falcon, the smaller this angle, which reduces the risk of control saturation, and thereby decreases the probability of missing the target.

## Discussion

Previous authors have suggested that falcons stoop at high-speed to add an element of surprise to their attack, thereby preventing escape maneuvers by the prey. Our simulations suggest that there are many other, previously unrecognized advantages to stooping: for example, a high-speed stoop still provides a clear advantage over an attack from lower altitudes even if the prey individuals fly erratically, which models how real prey behave when alerted to the presence of a predator. This is because fast-flying falcons can obtain a higher load factor, can roll faster into a turn, and can slow down less when increasing their load factor to maneuver—each of which increases the falcon’s catch success. Moreover, the steep angle of the stoop, and the details of the attack geometry of a fast-flying falcon further enhance catch success. The functional reasons for stooping are therefore far richer than considered previously, and are closely related to the physical constraints upon the problem (see [24] for a wider discussion of the operation of natural selection in relation to biomechanical constraint).

In order to intercept prey successfully at high speed, our model-falcons required a suitably optimized guidance law. Informed by a recent empirical study [15], our model-falcons used a pure proportional navigation guidance law, but we identified the optimal value of the navigation constant *N* post hoc through Monte Carlo simulation. A range of different values of *N* can be used successfully in low-speed flight, or when attacking non-maneuvering and smoothly-maneuvering prey, so the tightly-defined optimum of *N* ≈ 3 that applies in a high-speed stoop works well under all of the conditions that we tested (Fig 4). This most broadly effective value of the navigation constant coincides closely with the median value of *N* = 2.6 that has been found empirically in captive-bred peregrine falcons attacking artificial targets [15]. It also coincides with a classical result of linear-quadratic optimal guidance theory, which shows that proportional navigation with an effective navigation constant *N*′ = 3 minimizes the control effort needed to intercept a non-maneuvering target [14] (the effective navigation constant is defined as *N*′ = *N*(*v* cos *δ*)/*v*_*c*_ for pure proportional navigation, where *v* is the attacker’s speed relative to the ground, *v*_*c*_ its closing speed relative to the target, and *δ* the deviation angle between the attacker’s velocity vector and its line-of-sight to target [14]). The fact that a navigation constant of *N* ≈ 3 is found to be optimal or near-optimal in so wide a range of circumstances—and in so wide a range of systems, from birds to missiles—strongly indicates the robustness of our analysis and conclusions.

The results of our simulations also offer insight into the variability that is intrinsic to the attack behavior of real falcons. Captive-bred falcons have been shown to use a range of navigation constants around the median value of *N* = 2.6 that is comparable to the range of values of *N* that are optimal under the various conditions simulated in our model [15]. Whereas these real falcons seem to maintain an approximately constant value of *N* during a single interception attempt, they also vary it appreciably between attacks. It is currently unknown what drives this variability in the tuning of the navigation constant. Our model suggests that the details of the prey’s motion, and the details of the falcon’s flight speed, response delay, and precision in vision and control are all important determinants of the optimal tuning of *N*, and might therefore explain any adaptive variation in *N*.

Surprisingly, attacking at high speed does not require a faster response from the falcon than attacking at low speed (Fig 6e). By increasing the falcon’s flight performance, the stoop compensates for the decrease in catch success that would otherwise result from a slow response. In contrast, the need for accuracy in vision and control is especially acute when flying at high speed, and stooping is only optimal if the falcon has reasonably low error in both (Fig 6). Hence, we assume that selection for high visual acuity will be especially strong in species that use high-speed attacks. It follows that stooping should be considered a specialist hunting technique, because only accurate falcons with optimized guidance will be able to increase catch success by stooping. This arguably poses an exploration-exploitation dilemma for a falcon learning to catch prey: either it may seek to optimize its current catch success by adopting the easy strategy of a low-speed attack, for which the details of the parameter tuning are not critical; or, it may explore the more difficult strategy of a high-speed stoop, which could decrease catch success at first in an unskilled falcon, but can be expected to increase catch success in the long-run. The playful attacks by falcons in which they do not seriously attempt to kill their prey [6], may be necessary for acquiring sufficient skill in stooping.

### Limitations and wider implications

Although our physics-based model is realistic enough for its intended purpose, there are obviously further constraints in nature that we have not modelled here, including the effects of unsteady aerodynamics, the dynamics of pitch and yaw instability, and the mechanics of catching or knocking the prey with the talons at intercept. There are also other complicating factors that we have not modelled, including the effects of explicit evasive maneuvers by the prey, or the impact of intra- and inter-specific variation in flight morphology and physiology, and hence variation in the flight performance of predator or prey. These factors can be studied through extensions of the model and through parametric variation of the model between simulations, and will be considered elsewhere. Nevertheless, our approach to studying the dynamics of aerial predation is unique among behavioral studies of complex systems in combining guidance and control laws inspired by missile theory [14] with a detailed simulation model of the biology and physics of animal flight. The underlying feedback laws are well-founded in the theory of optimal guidance [14], and their validity as a phenomenological model of guidance and control in peregrine falcons has already been verified in nature [15]. Furthermore, the simulation approach that we have used proves necessary because of the complexity of the flight dynamics, which precludes an analytical approach [13]. Even setting aside the aerodynamic complexities that we have handled using a blade-element model of flapping flight, the mere fact that the birds must reorient their body to redirect their lift vector generates dynamics that are known to have no analytical solution in the most-closely analogous case of bank-to-turn missiles [25]. Our modeling therefore follows an embodiment approach, which states that behavior emerges through feedback-loops between the brain, the body, and the world. Aspects of cognition, such as the guidance laws used to intercept prey, are shaped by properties of the body and therefore bodily traits need to be considered to fully understand behavior [12, 26]. In summary, our agent-based simulation approach provides insights into the optimization of attack strategies by an aerial predator that could not have been reached in any other way, and thereby paves the way for a new generation of studies into the optimization of complex multi-agent flight behaviors.

## Materials and methods

### Summary of simulation procedure

In our simulations, model-birds fly with six degrees of freedom through an open three-dimensional space without objects or boundaries. In each simulation run, a model-falcon aims to intercept a lone model-starling in mid-air, using a pure proportional navigation guidance law (Eq 1). In model-starlings, the guidance command is a forcing function that ensures that they either fly linearly, or execute smooth or non-smooth maneuvers, always keeping within ±20m of their initial altitude. Model-birds are subject to gravitational and aerodynamic forces, and flap, glide and retract their wings to manipulate the aerodynamic forces. Model-birds maximize their forward acceleration at a given speed and orientation, subject to the constraint that they meet the normal acceleration commanded by their guidance system. A flight controller determines the changes in wing shape and motion that best meet the desired acceleration. When the commanded normal acceleration cannot be met, model-birds simply exert the maximum attainable lift force.

At the start of an attack, the model-starling is located at the origin of the global coordinate system, with its body coordinate system oriented randomly. This variation in initial orientation ensures sufficient randomization to avoid artificial results due to coupling of highly specific initial conditions of the falcon and starling. The model starling begins flying at an initial speed of 11 ms^−1^, calculated as the airspeed at which the cost of transport is minimized under the model. The model-falcon initially flies at a speed of 16 ms^−1^, with its longitudinal body axis pointing directly towards the starling, and its lateral body axis horizontal. We parametrically vary the falcon’s initial position relative to the prey, and vary the navigation constant *N* (i.e. the one free parameter of the falcon’s guidance law; see below) to simulate a continuum of different attack strategies. For each attack, we sample at random from a uniform distribution, sampling the navigation constant *N* between 1 and 20, the falcon’s initial altitude above the prey between −200 and 1500 m, and the initial horizontal distance to the prey between 0 and 800 m. The simulation ends when the falcon either intercepts the starling or is unsuccessful in its attempt to intercept, according to the criteria defined below. For a visualization of the simulations, see SI videos.

### Analysis

Every simulation ends in either the success or failure of the model-falcon to catch the model-starling. A catch is defined as occurring when the model-falcon comes within 0.2m of the model-starling. Failure occurs if either the falcon has not caught the starling within 40 s, or if it experiences a near-miss from which it cannot recover. A near-miss occurs when the model-falcon comes within 5.0 m of the model-starling, but subsequently finds itself further than this from the model-starling and with the model-starling in the blind zone of the model-falcon (a cone of 45° behind the bird) such that the falcon would effectively need to begin a new engagement in order to re-acquire its target. In order to analyze how the model parameters affect catch success, we apply Generalized Additive Modeling (GAM; [19, 20]). This is a nonlinear regression method which places no assumptions on the shape of the relationship between predictor and outcome. The estimation of the smoothing functions is conducted by automated cross-validation procedures (quadratic penalized likelihood), which reduce the likelihood of over-fitting and therefore ensure that our (conditional) maxima are not spurious. We applied GAMs with a logit link function, with catch success as the outcome variable and with the navigation constant *N*, *initial-altitude* and *horizontal-distance* as the independent variables. We built separate models for each combination of prey motion, response delay, and error. No constraints on the effective degrees of freedom were applied.

### Software

Model simulations were programmed in C++, using openGL for graphics rendering. Hildenbrandt’s StarDisplay model [27] was used as the framework for graphical display. Optimization studies of the blade-element model were conducted in MATLAB 2014a, and the *mgcv* package [28] of R statistics [29] was used for GAM regression.

### Detailed description of the bird-flight simulator

Here we explain the detail of our simulation model, using the block structure depicted in Fig 2, and discussing each of the following four segments of the block diagram in turn: A. Kinematics, B. Vision, C. Guidance, D. Control and E. Aerodynamics. We justify each variable and mechanism by parameterization to empirical data, and justify mathematical argument in terms of physics or optimality. For symbol meanings, see Table 2.

**Table 2:** Symbol meanings.

symbol	explanation
*α*	angle of attack
*β*	dive angle (negated elevation angle)
*ϵ*	visual error vector
*θ*	wing-beat angular amplitude
*κ*	altitude relative to preferred altitude
$\vec{\dot{\lambda }}$	angular velocity vector of the line-of-sight
$\vec{\mu }$	vector of morphological properties
*μ*	dynamic viscosity of air
*ξ*	limit of the distribution of visual error
*ρ*	air density
*τ*	response delay, differencing time of visual input and update interval
*ϕ*	fraction of wing retraction
*ω*	roll (angular) velocity
Ω	roll angle
$\vec{a}$	translational acceleration vector
${\vec{a}}_{steer}$	commanded acceleration vector
${\vec{a}}_{projected}$	acceleration vector outputted by the weight support controller
AR	aspect ratio
*B*	body coordinate system
*b*	wing span
*b*_*min*_	minimum wing span
*b*_*max*_	maximum wing span
*c*_*n*_	various constants in equations
*c*_*l*_	lift coefficient
*c*_*l*.*max*_	maximum lift coefficient
*c*_*torque*_	decrement in thrust due to torque
*c*_*d*.*fric*_	friction drag coefficient
*c*_*d*.*body*_	body drag coefficient
*c*_*d*.*induced*_	induced drag coefficient
*dt*	model time-step
${\vec{F}}_{flight}$	total translational force
*f*	wing-beat frequency
*g*	gravitational acceleration
*I*_*x*′_	total moment of inertia about the roll axis
*I*_*b*_	body inertia around the roll axis
*I*_*b*0_	body inertia for body width of 1m and body mass of 1kg
*I*_*wing*_	wing inertia around the shoulder
*I*_*wing*.*center*_	wing inertia around the center of gravity of the bird
*J*	sum of mass times distance to center of gravity
*L*	lift
*L*_0_	maximum lift at maximum wing span
*L*_*max*_	maximum attainable lift
*l*_*w*_	wing length
*M*_*x*′_	net torque around the roll axis
*M*_*wing*_	net torque around the roll axis of one wing
*m*	total mass
*m*_*w*_	wing mass
*m*_*b*_	body mass
$\vec{q}$	random unit vector
*Q*	random unit vector
$\vec{r}$	position vector
${\vec{\hat{r}}}_{d}$	estimated position of the starling relative to the falcon
*Re*	Reynolds number
*S*_*w*_	wing area
*S*_*b*_	frontal projected body area
*t*	time
TD	magnitude of thrust—drag
$\text{T}\;D_{f}$	magnitude of thrust—drag when flapping
$\text{T}\;D_{g}$	magnitude of thrust—drag when gliding
*U*	airspeed relative to wing motion
*U*_*a*_	airspeed
*U*_*w*_	speed of a single wing-blade
$\vec{v}$	velocity vector
${\vec{v}}_{falcon}$	velocity vector of falcon
${\vec{\hat{v}}}_{d}$	estimated velocity of the starling relative to the falcon
*v*_*thresh*_	threshold speed for torque constraints
*w*_*b*_	body width

#### A Kinematics

From a flight dynamics perspective, birds are implemented in the model as rigid bodies with six degrees of freedom. Model birds bank to turn, so as a simplification we take account only of their roll moment of inertia in modelling the rotational dynamics. The position vector $\vec{r}$ determines the position of the bird in a right-handed inertial axis system (*x*, *y*, *z*) in which ${\vec{e}}_{x}$, ${\vec{e}}_{y}$, ${\vec{e}}_{z}$ are unit vectors defining the inertial axes, where ${\vec{e}}_{z}$ points upward and therefore defines the bird’s altitude. The orientation of the bird is described by a rotating right-handed body-fixed axis system (*x*′, *y*′, *z*′), in which ${\vec{e}}_{x^{\prime }}$, ${\vec{e}}_{y^{\prime }}$, and ${\vec{e}}_{z^{\prime }}$ are unit vectors directed along the principal axes of the bird, and called the roll, pitch, and yaw axes, respectively. The roll axis ${\vec{e}}_{x^{\prime }}$ is assumed to be aligned instantaneously with the bird’s forward velocity $\vec{v}$, which amounts to assuming perfect pitch and yaw stability, and together with the yaw axis ${\vec{e}}_{z^{\prime }}$ is assumed to define the bird’s plane of bilateral symmetry.

The kinematics of the model are governed by Newton’s laws of motion. Numerical Verlet integration is used to solve the translational motion of the bird according to the following differential equations:

$$\begin{array}{c} \vec{r}\left(t+dt\right)=\vec{r}\left(t\right)+\vec{v}\left(t\right)dt+\frac{1}{2}\vec{a}\left(t\right)dt^{2} \end{array}$$ (2)

$$\begin{array}{c} \vec{v}\left(t+dt\right)=\vec{v}\left(t\right)+\frac{1}{2}\left(\vec{a}\left(t\right)+\vec{a}\left(t+dt\right)\right)dt \end{array}$$ (3)

$$\begin{array}{c} \vec{a}\left(t+dt\right)=\frac{\vec{F}}{m} \end{array}$$ (4)

where $\vec{r}$ is the position, $\vec{v}$ is the velocity, $\vec{a}$ is the acceleration, and *m* is the mass of the bird. The update time *dt* is the time step of the model at which both the numerical integration scheme and the flight forces are updated, and is set at 1 × 10^−4^ s (see section L for convergence tests). The flight force $\vec{F}$ is composed as follows:

$$\begin{array}{c} \vec{F}=\text{T}\;D{\vec{e}}_{x^{\prime }}+L{\vec{e}}_{z^{\prime }}-mg{\vec{e}}_{z} \end{array}$$ (5)

where TD is the net thrust minus drag force, *L* the lift force, and *g* the gravitational acceleration. The time-varying aerodynamic forces TD and *L* are outputted by the lift controller described in Section D.2. Model-birds respond to their normal acceleration command by reorienting their lift vector in a banked turn, applying lift asymmetrically so as to generate a net roll torque. To simplify the model, it is assumed that the exerted roll does not affect lift, thrust and drag. Wing retraction is assumed to be symmetric, so that asymmetric lift forces are produced only by angle of attack asymmetries, and the roll moment of inertia depends appropriately on the retraction of the wings. The rotation of the body about its roll axis is updated as follows:

$$\begin{array}{c} \Omega (t+dt)=\Omega (t)+\omega (t)dt \end{array}$$ (6)

$$\begin{array}{c} \omega \left(t+dt\right)=\omega \left(t\right)+\dot{\omega }\left(t\right)dt \end{array}$$ (7)

$$\begin{array}{c} \dot{\omega }=\frac{M_{x^{\prime }}}{I_{x^{\prime }}} \end{array}$$ (8)

where Ω is the roll angle, *ω* is the roll angular velocity, *I*_*x*′_ is the total moment of inertia of the bird about its roll axis ${\vec{e}}_{x^{\prime }}$, and *M*_*x*′_ is the roll torque. The time-varying roll inertia *I*_*x*′_ and roll torque *M*_*x*′_ are outputted by the roll controller described in Section D.3. Eq 8 assumes that the body axes (*x*′, *y*′, *z*′) are principal axes, and that no inertial coupling occurs.

#### B Vision

The model-falcon is assumed to apply a pure proportional navigation (PPN) guidance law, such that the function of its visual system is to estimate the angular rate of change $\vec{\dot{\lambda }}$ in the line-of-sight to target ${\vec{r}}_{d}={\vec{r}}_{f}-{\vec{r}}_{p}$, where ${\vec{r}}_{f}$ is the position of the falcon and ${\vec{r}}_{p}$ the position of its prey. In other words, the function of the visual system is to estimate the angular rate of change in the line drawn from the model-falcon to the model-starling, which, importantly, is independent of gaze direction. Mathematically, the line-of-sight rate is calculated as:

$$\begin{array}{c} \vec{\dot{\lambda }}=\frac{{\vec{\hat{r}}}_{d}\times {\vec{\hat{v}}}_{d}}{|{\vec{\hat{r}}}_{d}{|}^{2}} \end{array}$$ (9)

where ${\vec{\hat{r}}}_{d}$ is the line-of-sight vector measured with error, and where ${\vec{\hat{v}}}_{d}$ is the corresponding velocity of the falcon relative to its prey. Although we assume that the falcon holds an explicit representation of the line-of-sight rate $\vec{\dot{\lambda }}$, it is important to note that our use of Eq 9 does not imply that the bird must necessarily hold an explicit representation of its own position and velocity relative to its prey. For example, the line-of-sight rate on the lefthand side of Eq 9 could be estimated directly from the retinal coordinates of the target, provided that the falcon is able to subtract from this the apparent motion of the target due to rotation of its retinal coordinate system. Rather, Eq 9 should be thought of as providing a convenient way of computing the line-of-sight rate in the simulation, which simultaneously allows us to model the effects of error in the falcon’s visual system phenomenologically.

We incorporate this visual error by defining a random error vector $\vec{\epsilon }$ whose magnitude is drawn from the uniform distribution from 0 to *ξ* (see Section F for explanation and justification of the magnitude of *ξ*), and whose direction is drawn from the uniform circular distribution around the true line-of-sight vector ${\vec{r}}_{d}$. This visual error vector $\vec{\epsilon }$ explicitly models the effects of angular error in the estimation of the direction of the line-of-sight vector ${\vec{r}}_{d}$, and is therefore scaled by the target range $|{\vec{r}}_{d}|$ when computing the estimate of the line-of-sight vector required by Eq 9:

$$\begin{array}{c} {\vec{\hat{r}}}_{d}={\vec{r}}_{d}+\vec{\epsilon }\left|{\vec{r}}_{d}\right| \end{array}$$ (10)

We calculate the corresponding relative velocity as:

$$\begin{array}{c} {\vec{\hat{v}}}_{d}=\frac{{\vec{\hat{r}}}_{d}\left(t\right)-{\vec{\hat{r}}}_{d}\left(t-\tau \right)}{\tau } \end{array}$$ (11)

where *τ* is the differencing time, which we also take to represent the sampling rate from vision to guidance in the falcon.

#### C Guidance

Changes in the model-falcon’s velocity are commanded by a pure proportional navigation (PPN) guidance law. The input to this guidance law is the estimated line-of-sight rate $\vec{\dot{\lambda }}$ from Eq 9, whilst the output from the guidance system to the controller is the commanded acceleration ${\vec{a}}^{*}$. In three dimensions, the PPN guidance law is defined as:

$$\begin{array}{c} {\vec{a}}^{*}=N\vec{\dot{\lambda }}\times \vec{v} \end{array}$$ (12)

where $\vec{v}$ is the falcon’s velocity and *N* is a constant of proportionality called the navigation constant. The form of this equation is such that the acceleration ${\vec{a}}^{*}$ commanded under PPN guidance is always perpendicular to the falcon’s velocity vector $\vec{v}$.

The guidance system of the model-starlings is described by one of three different kinds of forcing-function: straight flight, smooth maneuvers, and non-smooth maneuvers (see also Fig 1).

*C.1 Straight flight.* In straight flight, acceleration is commanded on a constant heading drawn at random from a uniform circular distribution, and at a constant elevation drawn at random from a uniform distribution between ±2.5°. In combination with its random initial orientation, this forcing function causes the model-starling to turn towards an almost horizontal flight direction within 1 or 2 s of the start of the simulation, after which it flies in a straight line whilst maximizing its forward acceleration. The magnitude of the commanded acceleration ${\vec{a}}^{*}$ is such that its combination with gravitational acceleration does not exceed the starling’s maximum normal acceleration as calculated in Section D.1.

*C.2 Smooth maneuvers.* In smooth turning, centripetal acceleration is commanded to the bird’s left or right, with oscillations in the magnitude of the commanded acceleration specified according to a harmonic forcing function. This smooth forcing function is described by the following equation:

$$\begin{array}{c} {\vec{a}}^{*}=\vec{h}{\left(c_{1}\text{sin}c_{2}t+c_{1}\right)}^{c_{3}}a_{max}+c_{4}\kappa {\vec{e}}_{z} \end{array}$$ (13)

where *t* is the time in seconds, and *κ* is the difference between the current and initial altitude of the starling. The last term in this equation ensures that the starling always remains close to its starting altitude. The unit vector $\vec{h}$ is perpendicular to the model-starling’s velocity vector and is directed horizontally:

$$\begin{array}{c} \vec{h}=\frac{\vec{e_{x^{\prime }}}\times {\vec{e}}_{z}}{|\vec{e_{x^{\prime }}}\times {\vec{e}}_{z}|} \end{array}$$ (14)

The terms *c*_1_, …, *c*_4_ in Eq 13 were optimized by varying them parametrically (see Table 3 for optimized settings) so as to maximize the mean magnitude of the normal acceleration, subject to the constraints that: 1. the mean roll rate remains below 30 rad s^−2^; 2. the starling exerts accelerations equivalently in all directions (see S1 Fig); 3. the starling flies within bounds of ±20 m of its initial altitude. The starling does not exert maximal normal acceleration at each time step, because it slows down when maneuvering, thereby decreasing the maximum normal acceleration in a future time step. The optimized smooth forcing function results in high load factors (mean load factor: 3.4) and low roll accelerations (mean roll acceleration magnitude: 27 rad s^−2^).

**Table 3:** Model settings.

parameter	setting
*dt*	0.0001
*c*_1_	0.7
*c*_2_	3.5
*c*_3_	0.13
*c*_4_	1.5
*c*_5_	0.008
*c*_6_	0.99
*c*_7_	1.6
*c*_8_	0.1
*c*_9_	20

*C.3 Non-smooth maneuvers.* In non-smooth turning, acceleration of approximately constant magnitude is commanded in a stepwise fashion in a randomly varying direction close to the horizontal. The non-smooth function has the following equation:

$$\begin{array}{c} {\vec{a}}^{*}=\vec{q}c_{6}a_{max}+c_{7}\kappa {\vec{e}}_{z} \end{array}$$ (15)

where $\vec{q}$ is a random unit vector that is updated by the following equation:

$$\begin{array}{c} \vec{q}\left(t+dt\right)=\{\begin{array}{cc} \vec{q}\left(t\right), & \text{if}\;c_{5}<i∼U\left(0,1\right)\\ c_{8}\vec{q}\left(t\right)+\left(1-c_{8}\right)\vec{Q} & \text{otherwise} \end{array} \end{array}$$ (16)

where *U* denotes sampling from a uniform distribution, and where $\vec{Q}$ is a unit vector pointing towards a random azimuth angle and elevation between −*c*_9_ and *c*_9_ degrees. The parameters *c*_5_, ‥, *c*_9_ are optimized as for the smooth forcing function (see Table 3), with the modification that the non-smooth forcing function also aims to maximize roll acceleration. The non-smooth forcing function results in similarly high load factors to the smooth forcing function (mean load factor: 3.4), but involves much higher roll accelerations (mean roll acceleration magnitude: 2012 rad s^−2^).

#### D Control

The bird’s flight controller ensures that the acceleration commanded by the guidance law is achieved or approximated by making the appropriate adjustments to the wing motion and shape. The bird’s flight controller is subdivided into three subsystems determining weight support, lift control, and roll control, which we now discuss in turn.

*D.1 Weight support.* To achieve a change in velocity with the magnitude and direction of the commanded acceleration, gravitational acceleration needs to be considered at each time point. Specifically, the sum of the centripetal acceleration due to lift and the component of gravitational acceleration that is perpendicular to the bird’s velocity should equate the commanded centripetal acceleration. Hence, to determine the required magnitude and direction of the lift force, gravitational acceleration is first subtracted from the commanded acceleration:

$$\begin{array}{c} {\vec{a}}_{steer}={\vec{a}}^{*}-g{\vec{e}}_{z} \end{array}$$ (17)

then projected onto the plane defined by the bird’s transverse and dorsoventral axes, ${\vec{e}}_{y^{\prime }}$ and ${\vec{e}}_{z^{\prime }}$:

$$\begin{array}{c} {\vec{a}}_{projected}=[\begin{array}{c} \;0\\ {\vec{a}}_{steer}\cdot {\vec{e}}_{y^{\prime }}\\ {\vec{a}}_{steer}\cdot {\vec{e}}_{z^{\prime }} \end{array}] \end{array}$$ (18)

The magnitude of the resulting acceleration ${\vec{a}}_{projected}$ is then signalled to the lift controller (described in Section D.2), whilst its direction is signalled to the roll controller (described in Section D.3).

*D.2 Lift control.* Lift control is governed by the acceleration objective described in section E: maximize forward acceleration given current airspeed and body orientation, subject to the constraint that the lift needed to meet the guidance commands is exerted. This objective could be achieved in multiple ways, whether by varying the wingbeat kinematics when flapping, or by retracting the wings to reduce drag when gliding. In this section, the wing-beat averaged equations that determine which of these flight modes is selected (see section E for a derivation of these equations).

The magnitude of the desired lift L* is $m|{\vec{a}}_{projected}|$. Constraints on the achievable lift arise physiologically due to due to constraints on the torque forces that the flight muscles can sustain, and aerodynamically due to wing stall, where *c*_*l*.*max*_ is the lift coefficient at the stall limit. To calculate the maximum achievable lift for which the muscle torque constraints are not exceeded, we use the allometric scaling rule *L*_0_ = 1.7*mg*, where *L*_0_ is the maximum lift at maximum wing span [11] (see section G for a justification of the mechanical constraints in our model). In flapping flight, we assume no wing retraction, and thus *L*_*max*_ = *L*_0_ is the upper limit. In gliding flight, model-birds retract their wings, and the maximum achievable lift *L*_*max*_ is found by [11]:

$$\begin{array}{c} b_{ml}=\left(b_{max}-b_{min}\right){\left(\frac{L_{0}}{\frac{1}{2}S_{w}c_{l.max}\rho v^{2}}\right)}^{1/2}+b_{min} \end{array}$$ (19)

$$\begin{array}{c} S_{w.ml}=S_{w.max}\frac{b_{ml}-b_{min}}{b_{max}-b_{min}} \end{array}$$ (20)

$$\begin{array}{c} L_{max}=\frac{1}{2}\rho S_{w.ml}c_{l.max}v^{2} \end{array}$$ (21)

where *b*_*ml*_ is the wing span that maximizes lift, *b*_*max*_ and *b*_*min*_ are the minimum and maximum wing span respectively, *S*_*w*.*max*_ is the wing area at maximum span, and *S*_*w*.*ml*_ is the wing area that maximizes lift, *ρ* is the air density, and *v* is the airspeed. The achievable lift *L* is the lower value of the desired lift L* and the maximum achievable lift *L*_*max*_. The following equations relate the achievable lift *L* to the maximum net thrust (or minimum drag):

$$\begin{array}{c} c_{l}=\frac{2L}{S_{w}\rho v^{2}} \end{array}$$ (22)

$$\begin{array}{c} \text{T}\;D=(1-\frac{c_{l}^{2}}{c_{l.max}^{2}})\frac{\pi^{3}}{16}\rho b^{2}{\text{sin}}^{2}\left(0.5\theta \right)f^{2}l_{w}^{2}c_{torque}-(c_{d.body}S_{b}+c_{d.fric}S_{w}+\frac{c_{l}^{2}S_{w}}{2\pi A\;R})\rho v^{2} \end{array}$$ (23)

where *c*_*l*_ is the wingbeat-averaged lift coefficient, *c*_*l*.*max*_ is the maximum achievable wingbeat-averaged lift coefficient, *c*_*d*.*fric*_ is the friction drag coefficient on the wing, *c*_*d*.*body*_ is the total drag coefficient of the body, *S*_*w*_ is the maximum projected wing area, and *S*_*b*_ is the frontally projected body area, AR is the wing aspect-ratio, *b* is the actual wing span, *θ* is the stroke angle from the highest point of the wing-beat to the lowest point, *f* is the wingbeat frequency, and *l*_*w*_ is the wing length. The coefficient *c*_*torque*_ accounts for the constraints in torque forces that the flight muscles can sustain. The torque forces increase linearly with airspeed *v*, for a given wing-beat frequency and optimal wing-twist, and exceed the sustainable threshold at a point *v*_*thresh*_ which is determined through simulation of the blade-element model described in section E (note that this expression is almost equivalent to stating that the maximum available power for flight is constant across values of airspeed). The coefficient *c*_*torque*_ is calculated as:

$$\begin{array}{c} c_{torque}=\min(1,\frac{v_{thresh}}{v}) \end{array}$$ (24)

It is important to note that the wingbeat-averaged lift coefficient *c*_*l*_ can differ substantially from the local lift coefficient at a particular section of the wing at a particular time point in the wingbeat. For instance, when the downstroke delivers an upward lift force, and the upstroke a downward lift force of equal magnitude, *c*_*l*_ will be zero. When the bird is flapping, *f* is set to the maximum observed wingbeat frequency for the species, jointly with a stroke amplitude derived using allometric scaling rules (see Table 4). When the bird glides, *f* = 0 by definition, and the wing span *b* is optimized to achieve *L* with the lowest amount of drag. The wing span *b* determines the wing area according to the relationship:

$$\begin{array}{c} S_{w}=S_{w.max}\frac{b-b_{min}}{b_{max}-b_{min}} \end{array}$$ (25)

and determines the aspect ratio as:

$$\begin{array}{c} A\;R=\frac{b^{2}}{S_{w}} \end{array}$$ (26)

**Table 4:** Morphological parameters of the bird species in the model.

parameter	symbol	peregrine falcon		common starling	
wingbeat frequency (hz)	f	5.1	^a^ [51]	10.5	[52]
wing length (m)	*l*_*w*_	0.284	[51]	0.185	[43]
wing span (m)	b	0.873	[51]	0.39	[43]
body mass (kg)	*m*_*b*_	528 ⋅ 10^−3^	[51]	70 ⋅ 10^−3^	[43]
wing mass (kg)	*m*_*w*_	32 ⋅ 10^−3^	^b^ [43]	3.7 ⋅ 10^−3^	[43]
body inertia (kg m^2^)	*I*_*b*_	448.0 ⋅ 10^−6^	^c^	15.65 ⋅ 10^−6^	^c^
extended wing inertia (*kg* *m*^2^)	*I*_*wing*_	296.6 ⋅ 10^−6^	^d^	14.55 ⋅ 10^−6^	^d^
wing summation term Eq 32	J	2501.3 ⋅ 10^−6^	^d^	152.8 ⋅ 10^−6^	^d^
wing area (m^2^)	*S*_*w*_	89.7 ⋅ 10^−3^		24.14 ⋅ 10^−3^	
wing aspect ratio	AR	8.49	[51]	6.40	
angular flapping amplitude (rad)	*θ*	0.4*π*	^e^	0.47*π*	^g^
body area (m^2^)	*S*_*b*_	4.275 ⋅ 10^−3^	^f^	2.1 ⋅ 10^−3^	[53]
body drag coefficient	*c*_*body*_	0.14	[44]	0.24	^h^ [53]
wing friction drag coefficient	*c*_*fric*_	14 ⋅ 10^−3^	^i^	9.35 ⋅ 10^−3^	^i^
speed at which torque constrains thrust (ms^−1^)	*v*_*thresh*_	16.5		11.62	
maximum steady lift coefficient	*c*_*l*.*max*_	1.6	[44]	1.6	[44]

^a^ maximum from range in Table 5 of [51]

^b^ estimated from [43] by interpolating between allometrically similar birds

^c^ see Eq 82 for the allometric scaling law applied

^d^ recalculated for a peregrine falcon and common starling, assuming mass distrubitions along the wing as in [30, 41]

^e^ estimated by analyzing slow-motion videos of peregrine falcons

^f^ allometric scaling law in Eq 80

^g^ log_10_ (*θ*/180) = 1.83–0.24 log_10_ (*b*) [54]

^h^ Literature estimates range from 0.2 to 0.35 and depend strongly on the measurement of the frontally projected area. We have taken the measurements of both variables from the same paper [53].

^i^ Friction drag depends on the Reynolds number (see section J).

To find the optimal wing span b* that achieves a given lift *L* with the least amount of drag, the following steps are applied. First b* is calculated by:

$$\begin{array}{c} b^{*}=\sqrt[{3}]{\frac{\left(b_{max}-b_{min}\right)8{\left(L^{*}\right)}^{2}}{\pi c_{d.fric}S_{w}{\left(\rho v^{2}\right)}^{2}}} \end{array}$$ (27)

(see Section J for derivation), and then b* is truncated if required to fall within the closed interval [*b*_*min*_, *b*_*max*_]. An additional upper bound on b* is the wingspan *b* at which the torque constraint is exceeded when exerting *L*:

$$\begin{array}{c} b^{*}=\max\left(b_{min},\min\left(b^{*},b_{max},L_{0}b_{max}/L\right)\right) \end{array}$$ (28)

*c*_*l*_ is subsequently calculated by Eq 22. Again, *c*_*l*_ is truncated not to exceed *c*_*l*.*max*_ and if truncated, b* is recalculated as:

$$\begin{array}{c} b^{*}=\frac{2L(b_{max}-b_{min})}{S_{w}c_{l.max}\rho v^{2}}+b_{min} \end{array}$$ (29)

and b* is truncated again to fall within the closed interval [*b*_*min*_, *b*_*max*_]. The bird chooses either to flap or to glide according to which mode of flight achieves or most closely approximates L* with the largest net thrust (or least drag).

*D.3 Roll control.* The roll controller determines the roll acceleration used to reach the desired bank angle. The desired direction of roll by the bird is determined by calculating the angle between *a*_*projected*_ and ${\vec{e}}_{z^{\prime }}$:

$$\begin{array}{c} \gamma ={\text{sin}}^{-1}(\frac{|{\vec{a}}_{projected}\times {\vec{e}}_{z^{\prime }}|}{|{\vec{a}}_{projected}|}) \end{array}$$ (30)

To roll in this direction, the bird must apply the appropriate roll acceleration. As Eq 8 implies, roll acceleration is determined by the the aerodynamic roll torque *M*_*x*′_ and the roll moment of inertia *I*_*x*′_. The inertia is dependent on the wing span *b*, whilst the net torque depends on the differential lift exertion between left and right wings, together with the wing span. Therefore, the goal of the roll controller is to find the optimal wing span and lift exertion to reach the desired bank angle in the minimum amount of time. We assume that on average the lift force acts at half the wing length, thus the total torque *M* generated by one wing is:

$$\begin{array}{c} M_{wing}=L_{wing}\frac{b}{4} \end{array}$$ (31)

The roll moment of inertia is calculated as:

$$\begin{array}{c} I_{x^{\prime }}=I_{b}+2\left(I_{wing}\phi^{2}+2.4\cdot 10^{-3}m_{b}^{0.70}m_{wing}+0.098m_{b}^{0.35}J\phi \right) \end{array}$$ (32)

(see section H for justification), where *I*_*x*′_ is the total moment of inertia about the roll axis, *I*_*b*_ is the body inertia, *I*_*wing*_ the inertia of one wing around the shoulder, *ϕ* the proportion of wing retraction, *m*_*w*_ the mass of the wing, *m*_*b*_ the body mass, and *J* = ∑_*i*_ *m*_*i*_ *r*_*i*_, where *r* denotes the distance from the bird’s shoulder when the wing is fully extended and where *m*_*i*_ denotes the mass of a blade element of the wing at that point.

To obey the guidance law, the bird must roll until it reaches the desired bank angle, as dictated by the guidance algorithm, and must then stop rolling. The faster the bird reaches the desired bank angle, the more accurately it obeys its guidance law. The fastest way to reach a given bank angle is to accelerate maximally until some point *q*, and then decelerate maximally (called Bang-Bang control). The highest roll acceleration is achieved at the wing span that maximizes lift production (see section I for a proof). Given this maximal acceleration, the bird must know this point *q* in the roll at which it should start to decelerate in order to reach a roll velocity of zero at the desired bank angle. We calculate *q* as follows. Let *ω* be the roll (angular) velocity and *γ* (see Eq 30) be the angular position relative to the desired bank angle. The angular acceleration $\dot{\omega }$ is either the positive or negative maximal angular acceleration. The angular velocity is

$$\begin{array}{c} \omega =\dot{\omega }t+\omega_{0} \end{array}$$ (33)

$$\begin{array}{c} \gamma =\frac{\dot{\omega }t^{2}}{2}+\omega_{0}t \end{array}$$ (34)

where *ω*_0_ is the angular velocity at the current time step. We calculate the time *t* it takes to reach *γ* by solving

$$\begin{array}{c} t=\frac{-\omega_{0}\pm \sqrt{\omega_{0}^{2}+2\dot{\omega }\gamma }}{\dot{\omega }} \end{array}$$ (35)

where the minimum positive *t* is the answer. If *q* (the point at which the bird should start to decelerate) is exceeded, there are 2 positive solutions for Eq 35, according to whether *a* has the opposite or same sign as *γ* (which is the case when turning towards the desired bank angle and decelerating). If the bird has not yet reached *q*, then there are no real solutions. These equations have no real solutions when:

$$\begin{array}{c} \omega_{0}^{2}+2\dot{\omega }\gamma <0 \end{array}$$ (36)

Thus the following rule obeys Bang-Bang control: $\dot{\omega }$ should have the same sign as *γ*, unless both *ω* has the same sign as *γ* and $\omega_{0}^{2}>2\dot{\omega }\gamma$, in which case $\dot{\omega }$ should have the opposite sign. When $\omega_{0}^{2}>2\dot{\omega }\gamma$, the equations have real and positive solutions when decelerating, which means that *γ* is reached within a positive time. The first moment in time when this is the case must be closest to the point *q*. Finally, the output of the controllers is fed to the kinematics, which completes the feedback loop.

#### E Aerodynamics

**Fig 7:**
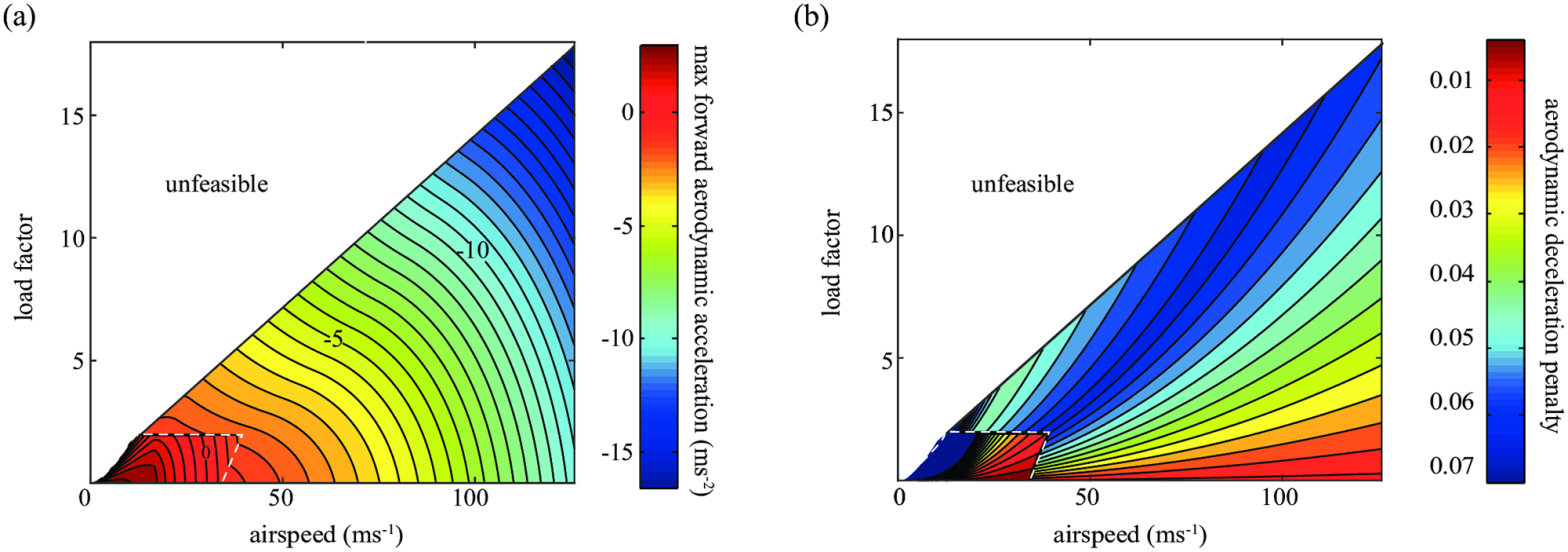
(a) Look-up table for the accelerations due to the aerodynamic forces acting on the falcon. At each model time-step, the falcon maximizes forward acceleration (minimizes deceleration), given its forward speed and with the constraint that load factor is set to achieve the net commanded acceleration by the guidance law. If this constraint cannot be met (i.e. if it is unfeasible due to aerodynamics or high resulting torque forces), the closest approximation of the load factor is chosen. In the blade-element model, the falcon optimizes the wing twist, the wing’s angle-of-attack, the wingbeat frequency and the wing retraction. Inside the trapezoidal contour, the falcon flaps at maximal wingbeat frequency, and outside it the falcon glides. Above the contour, flapping results in too high torque forces on the wing. Gravity is excluded from the accelerations in the figure. (b) The partial derivative of forward aerodynamic acceleration with respect to load factor, termed the “aerodynamic deceleration penalty”.

We used a multistage modeling approach to model the aerodynamic forces on the birds. First, we constructed a blade-element model of flapping and gliding flight, assuming sinusoidal wingtip kinematics and quasi-steady flow [30–36]. Since our model-birds mostly flew at low Strouhal numbers (St < 0.22), this quasi-steady analysis provides sufficient accuracy for our modeling objectives [31, 37]. We assumed that the relation between the lift coefficient and induced drag coefficient was that of an elliptical wing under classical lifting-line theory [38], and incorporated realistic physical constraints on the maximum aerodynamic torque, to account for the limits of sustained force production by the pectoralis and supracoracoideus [39, 40]. All of the morphological parameters needed to model body, friction, and induced drag, as well as lift and thrust were derived from empirical estimates [11, 30, 41–45], or estimated using allometric scaling laws. We then used a combination of regression statistics and analytic methods to derive a set of equations relating the maximum forward acceleration over a wingbeat to the bird’s load factor (i.e. lift divided by body weight) and airspeed (Fig 7a). To determine the maximum forward acceleration for a given load factor and airspeed, we optimized the time-varying wing twist and mean angle of attack, as well as the stroke plane, and whether the bird should flap at the maximum wingbeat frequency or glide and retract its wings. To account for errors in control, we modified the load factor by a multiplicative error term (*χ*) proportional to the rate of change in the lift coefficient, setting *χ* = 0 in our baseline model. Here, we present a derivation of the time-averaged equation (Eq 23) that we used in section D.

The goal is to come up with a simple approximate function

$$\begin{array}{c} \text{T}\;D=f(L,v,\vec{\mu }) \end{array}$$ (37)

Where TD is the time-averaged maximum thrust minus drag, *L* is the lift, *v* the airspeed, and $\vec{\mu }$ is a vector of morphological parameters that can be gathered empirically.

*E.1 Assumptions.* We assume the following aerodynamic and kinematic properties. For each blade-element the equations for lift and drag are:

$$\begin{array}{c} L\left(i,p\right)=\frac{1}{2}\rho c_{l}S\left(i\right)U{\left(i,p\right)}^{2} \end{array}$$ (38)

$$\begin{array}{c} D\left(i,p\right)=\frac{1}{2}\rho c_{d}S\left(i\right)U{\left(i,p\right)}^{2} \end{array}$$ (39)

Where *S*(*i*) denotes the total area of the element *i*, and *U*(*i*, *p*) is the speed of air relative to the (moving) blade at point *p* of the wing-beat cycle. $\vec{L}\left(i,p\right)$ is defined to be perpendicular to $\vec{U}\left(i,p\right)$ in the plane spanned by the blade, while $\vec{D}\left(i,p\right)$ is parallel to $\vec{U}\left(i,p\right)$ and has the opposite sign. The drag coefficient *c*_*d*_ is composed of the body, induced and wing-friction drag coefficients:

$$\begin{array}{c} c_{d}=c_{d.body}+c_{d.induced}+c_{d.friction} \end{array}$$ (40)

where the body drag coefficient *c*_*d*.*body*_ is referenced to the body’s frontal projected area *S*_*b*_. The induced drag coefficient *c*_*d*.*induced*_ is related to *c*_*l*_ by [46]:

$$\begin{array}{c} \frac{c_{d.induced}}{c_{l}}=\frac{c_{l}}{\pi A\;R} \end{array}$$ (41)

which is the classical limit from lifting line theory for a wing with an elliptical lift distribution, and we apply Blasius’ solution for laminar boundary layer of a flat plate [47] to obtain the friction drag coefficient:

$$\begin{array}{c} c_{d.friction}=2(\frac{1.328}{\sqrt{Re}}) \end{array}$$ (42)

where *Re* denotes the Reynolds number, which is calculated as

$$\begin{array}{c} Re=\frac{\rho U}{\mu }\sqrt{\frac{S}{A\;R}} \end{array}$$ (43)

with *μ* the dynamic viscosity of the air. Furthermore, the lift coefficient *c*_*l*_ is related to the angle of attack *α* by the higher-order lifting line theory [24]:

$$\begin{array}{c} c_{l}=\frac{2\pi \alpha }{1+2/A\;R+16\left(\log\pi A\;R-9/8\right)/{\left(\pi A\;R\right)}^{2}} \end{array}$$ (44)

We further assume that the wing shape is elliptical, that there is no side-slip, that the airfoil is symmetric and that the wing-motion is sinusoidal.

*E.2 Optimization problem.* Our aim is to find the function $c_{l}^{*}\left(i,p\right)$ that maximizes TD under several constraints. Because the friction drag and body drag are independent of *c*_*l*_, we maximize $\text{T}\;D_{i}$, which is thrust minus induced drag. The objective function is a double integral over the wing length *i* and wing-beat cycle *p*:

$$\begin{array}{c} \text{T}\;D_{i}=\max_{c_{l}\left(i,p\right)}\int_{p=0}^{2\pi }2\int_{i=0}^{l_{w}}c_{1}\left(i,p\right)c_{l}\left(i,p\right)-c_{2}\left(i,p\right)c_{l}{\left(i,p\right)}^{2}didp \end{array}$$ (45)

with the equality constraint that lift equals the desired lift:

$$\begin{array}{c} L*=\int_{p=0}^{2\pi }2\int_{i=0}^{l_{w}}c_{3}\left(i,p\right)c_{l}\left(i,p\right)+c_{4}\left(i,p\right)c_{l}{\left(i,p\right)}^{2}didp \end{array}$$ (46)

and the inequality constraints

$$\begin{array}{c} -c_{l.max}<c_{l}\left(i,p\right)<c_{l.max},\forall \left(p,i\right):p\in \left\{0,2\pi \right\},i\in \left\{0,1\right\} \end{array}$$ (47)

$$\begin{array}{c} -t_{max}<\int_{i=0}^{l_{w}}ic_{3}\left(i,p\right)c_{l}\left(i,p\right)+ic_{4}\left(i,p\right)c_{l}{\left(i,p\right)}^{2}di<t_{max},\forall p\in \left\{0,2\pi \right\} \end{array}$$ (48)

where *c*_*l*.*max*_ is the lift coefficient at the stall limit, *t*_*max*_ is the maximum torque that the flight muscles can sustain, and where

$$\begin{array}{c} c_{1}\left(i,p\right)=\frac{U_{w}\left(i,p\right)}{2\sqrt{U_{w}{\left(i,p\right)}^{2}+U_{a}^{2}}}S\rho U^{2} \end{array}$$ (49)

$$\begin{array}{c} c_{2}\left(i,p\right)=\frac{U_{a}}{2\sqrt{U_{w}{\left(i,p\right)}^{2}+U_{a}^{2}}\pi A\;R}S\rho U^{2} \end{array}$$ (50)

$$\begin{array}{c} c_{3}\left(i,p\right)=\frac{U_{a}}{2\sqrt{U_{w}{\left(i,p\right)}^{2}+U_{a}^{2}}}S\rho U^{2} \end{array}$$ (51)

$$\begin{array}{c} c_{4}\left(i,p\right)=\frac{U_{w}\left(i,p\right)}{2\sqrt{U_{w}{\left(i,p\right)}^{2}+U_{a}^{2}}\pi A\;R}S\rho U^{2} \end{array}$$ (52)

where *U*_*w*_(*i*, *p*) is the speed of blade-element *i* in section *p* of the wing-beat cycle, *U*_*a*_ is the airspeed and $U^{2}=U_{w}{\left(i,p\right)}^{2}+U_{a}^{2}$. To start solving this objective function, we make a few observations. First, the unconstrained optimal $c_{l}^{*}\left(i,p\right)$ can be written as

$$\begin{array}{c} c_{l}^{*}\left(i,p\right)=\frac{U_{w}\left(i,p\right)}{2U_{a}}\pi A\;R=\frac{U_{w}\left(i,p\right)}{2U_{a}}c_{0} \end{array}$$ (53)

and when *U*_*a*_ ≫ *U*_*w*_(*i*, *p*), the value of the unconstrained objective function remains constant with respect to changes in airspeed and $c_{l}^{*}\left(i,p\right)$ converges to zero. Also, because the wings are symmetric, the maximum of the unconstrained $\text{T}\;D_{i}$ always corresponds to *L* = 0. Therefore, to get the maximum value of the unconstrained $\text{T}\;D_{i}$, we solve for $\lim_{U_{a}\to \infty }\text{T}\;D_{i}$. First, we rewrite the equation:

$$\begin{array}{c} \text{T}\;D_{i}=\left(\frac{U_{w}}{\sqrt{U_{w}^{2}+U_{a}^{2}}}\frac{U_{w}}{2U_{a}}c_{0}-\frac{U_{a}}{\sqrt{U_{w}^{2}+U_{a}^{2}}}\frac{U_{w}^{2}}{2^{2}U_{a}^{2}}c_{0}\right)c_{5}\left(U_{w}^{2}+U_{a}^{2}\right) \end{array}$$ (54)

where $c_{5}=\frac{1}{2}S\rho$. Removing all brackets:

$$\begin{array}{c} \text{T}\;D_{i}=\frac{c_{0}c_{5}U_{w}^{4}+c_{0}c_{5}U_{w}^{2}U_{a}^{2}}{2U_{a}\sqrt{U_{w}^{2}+U_{a}^{2}}}-\frac{c_{0}c_{5}U_{a}U_{w}^{4}+c_{0}c_{5}U_{a}^{3}U_{w}^{2}}{4U_{a}^{2}\sqrt{U_{w}^{2}+U_{a}^{2}}} \end{array}$$ (55)

We can note that:

$$\begin{array}{c} \lim_{U_{a}\to \infty }U_{a}\sqrt{U_{w}^{2}+U_{a}^{2}}=U_{a}^{2} \end{array}$$ (56)

and

$$\begin{array}{c} \lim_{U_{a}\to \infty }U_{a}^{2}\sqrt{U_{w}^{2}+U_{a}^{2}}=U_{a}^{3} \end{array}$$ (57)

Therefore

$$\begin{array}{c} \text{T}\;D_{i.max}=\lim_{U_{a}\to \infty }T=\frac{1}{2}c_{0}c_{5}U_{w}^{2}-\frac{1}{4}c_{0}c_{5}U_{w}^{2}=\frac{1}{4}c_{0}c_{5}U_{w}^{2} \end{array}$$ (58)

Thus

$$\begin{array}{c} \text{T}\;D_{i.max}=\frac{\pi }{8}A\;R\rho SU_{w}^{2} \end{array}$$ (59)

which is

$$\begin{array}{c} \text{T}\;D_{i.max}=\frac{\pi }{8}\rho b^{2}n\left(i\right)U_{w}{\left(i,p\right)}^{2} \end{array}$$ (60)

where *n*(*i*) is the fraction of the total wing area. Next, numerical simulations show that, despite the non-linearities caused by the inequality constraints, we can accurately fit a quadratic model of the form:

$$\begin{array}{c} \text{T}\;D_{i}=aL^{2}+b \end{array}$$ (61)

Also, our numerical simulations show that, when *L* is maximized, $\text{T}\;D_{i}$ by flapping converges to $\text{T}\;D_{i}$ by gliding. Therefore, we know two properties of the quadratic function:

$$\begin{array}{c} \text{T}\;D_{i}\left(L_{max},v,\vec{\mu }\right)=\text{T}\;D_{i}\left(c_{l.max}\frac{1}{2}S\rho v^{2},v,\vec{\mu }\right)=-c_{l.max}^{2}\frac{1}{2\pi A\;R}S\rho v^{2} \end{array}$$ (62)

$$\begin{array}{c} \text{T}\;D_{i}\left(L_{min},v_{max},\vec{\mu }\right)=\text{T}\;D_{i}\left(0,\infty ,\vec{\mu }\right)=\text{T}\;D_{i.max} \end{array}$$ (63)

Because $\text{T}\;D_{i}=\text{T}\;D_{i.max}$ when *L* = 0, we know that $b=\text{T}\;D_{i.max}$. From Eq 62 we know that:

$$\begin{array}{c} -c_{l.max}^{2}\frac{1}{2\pi A\;R}S\rho v^{2}=aL^{2}+\text{T}\;D_{i.max}=a{\left(c_{l.max}\frac{1}{2}S\rho v^{2}\right)}^{2}+\text{T}\;D_{i.max} \end{array}$$ (64)

Therefore *a* is:

$$\begin{array}{c} a=\frac{-2}{\pi A\;RS\rho v^{2}}-\frac{\text{T}\;D_{i.max}}{{\left(c_{l.max}\frac{1}{2}S\rho v^{2}\right)}^{2}} \end{array}$$ (65)

The relation between thrust and lift becomes:

$$\begin{array}{c} \text{T}\;D_{i}=(\frac{-2}{\pi A\;RS\rho v^{2}}-\frac{\text{T}\;D_{i.max}}{{\left(c_{l.max}\frac{1}{2}S\rho v^{2}\right)}^{2}})L^{2}+\text{T}\;D_{i.max} \end{array}$$ (66)

which can also be written as:

$$\begin{array}{c} \text{T}\;D_{i}=\frac{-cl^{2}S\rho v^{2}}{2\pi A\;R}+(1-\frac{cl^{2}}{c_{l.max}^{2}})\frac{\pi }{8}\rho b^{2}n\left(i\right)U_{w}{\left(i,p\right)}^{2} \end{array}$$ (67)

All that is left to do is to compute the double integral over the wing span and wing-beat cycle:

$$\begin{array}{c} \int_{p=0}^{1}\int_{i=0}^{1}n\left(i\right)U_{w}{\left(i,p\right)}^{2}didp \end{array}$$ (68)

Assuming sinusoidal flapping, the position (height) of the wing section *i* in time is determined by

$$\begin{array}{c} \text{sin}\left(\frac{1}{2}\theta \right)\text{sin}\left(2\pi ft\right)l_{w}i \end{array}$$ (69)

where *t* denotes the time. Differentiating this with respect to *t*, we get the speed of the wing section over time

$$\begin{array}{c} f2\pi \text{sin}\left(\frac{1}{2}\theta \right)\text{cos}\left(2\pi ft\right)l_{w}i \end{array}$$ (70)

Assuming an elliptical wing, the double integral is:

$$\begin{array}{l} \frac{1}{\pi }\int_{p=0}^{1}\int_{i=0}^{1}\sqrt{1-i^{2}}{\left(f2\pi \text{sin}\left(\frac{1}{2}\theta \right)\text{cos}\left(2\pi p\right)l_{w}i\right)}^{2}didp=\\ \;\pi {\left(l_{w}f2\text{sin}\left(\frac{1}{2}\theta \right)\right)}^{2}\int_{p=0}^{1}{\text{cos}}^{2}\left(2\pi p\right)\int_{i=0}^{1}\sqrt{1-i^{2}}{\left(i\right)}^{2}didp \end{array}$$ (71)

We first compute the inner integral:

$$\begin{array}{c} \int_{i=0}^{1}\sqrt{1-i^{2}}{\left(i\right)}^{2}di=\frac{{\text{sin}}^{-1}i}{2}-\sqrt{1-i^{2}}\left(\frac{i}{2}-i^{3}\right) \end{array}$$ (72)

and evaluated at *x* = 1, we get:

$$\begin{array}{c} \frac{\pi }{4} \end{array}$$ (73)

We thus need to solve

$$\begin{array}{c} {\left(f\pi \text{sin}\left(\frac{1}{2}\theta \right)l_{w}\right)}^{2}\int_{0}^{1}{\text{cos}}^{2}\left(2\pi p\right)dp \end{array}$$ (74)

The solution of the integral is

$$\begin{array}{c} \int_{0}^{1}{\text{cos}}^{2}\left(2\pi p\right)dp=\frac{p}{2}+\frac{\text{sin}(4\pi p)}{42\pi } \end{array}$$ (75)

And evaluated at 1 and 0, we get:

$$\begin{array}{c} \frac{1}{2}+\frac{\text{sin}(4\pi )}{8\pi }-\frac{1}{8\pi } \end{array}$$ (76)

Thus the solution of the double integral is

$$\begin{array}{c} {\left(f\pi \text{sin}\left(\frac{1}{2}\theta \right)l_{w}\right)}^{2}\left(\frac{1}{2}+\frac{\text{sin}(4\pi )}{8\pi }-\frac{1}{8\pi }\right) \end{array}$$ (77)

Of which by far the most significant factor is the first term. Keeping only the first term, we get

$$\begin{array}{c} \int_{p=0}^{1}\int_{i=0}^{1}n\left(i\right)U_{w}{\left(i,p\right)}^{2}didp\approx \frac{{\left(f2\pi \text{sin}\left(\frac{1}{2}\theta \right)l_{w}\right)}^{2}}{4}\frac{1}{2}=\frac{1}{2}{\left(f\pi \text{sin}\left(\frac{1}{2}\theta \right)l_{w}\right)}^{2} \end{array}$$ (78)

Thus the final thrust becomes:

$$\begin{array}{c} \text{T}\;D_{i}=\frac{-c_{l}^{2}S\rho v^{2}}{2\pi A\;R}+\left(1-\frac{c_{l}^{2}}{c_{l.max}^{2}}\right)\frac{\pi^{3}}{16}\rho b^{2}{\text{sin}}^{2}\left(0.5\theta \right)f^{2}l_{w}^{2} \end{array}$$ (79)

Subsequently, the coefficient for the decrement in thrust due to torque is added, as well as the friction and body drag, to produce Eq 23.

#### F Justification of the bounds on visual error

In the absence of any detailed information on visual processing in falcons, we estimate the magnitude of visual error by the falcon in the following way. Hodos *et al* [48] found that the minimum retinal image velocity for the detection of motion in pigeons is about 8°s^−1^. We assume that this detection threshold corresponds to the point at which the apparent motion due to target motion exceeds the apparent motion due to random noise. We further assume that the differentiation time of the falcon is 50ms and that the apparent motion due to target motion must exceed the apparent motion due to random noise in this short time frame. Therefore, the retinal image must move at least 0.007 rads per 50ms to be perceived. We thus take this value as the bound *ξ* on the visual error (see Section B). We acknowledge that this method is somewhat arbitrary, but consider that stating it in this way helps to provided confidence that the assumed numerical bound on the visual error is likely to be of about the right order of magnitude.

#### G Justification of the mechanical constraints

Whether a bird is flapping or gliding, aerodynamic loads place strain on the muscles, ligaments, and bones, in particular when the bird is performing maneuvers. In our simulations, the constraints on the production of aerodynamic forces are such that the muscles, ligaments and bones would not break or tear in real birds, and that the power required to produce these forces would not exceed the muscle power available to counter the resulting torque. Here, we justify that we only need to consider the constraint on the torque that arises around the shoulder due to lift production, as this constraint is likely to be exceeded before any other constraint.

The torque around the shoulder needs to be countered by the flight musculature, including in particular the pectoralis and supracoracoideus muscles. Bayer *et al*. [39], provide detailed measurements of the forces and constraints on these muscles in gliding flight of the domestic pigeon *Columba livia*. We use their measurements to verify Pennycuick’s [40] and Tucker’s [11] proposal for a general allometric scaling law for maximum lift production in birds. In horizontal gliding flight, lift balances weight (*mg*). Therefore, each wing carries half the body weight, which we assume to be applied at half the total wing length (*l*_*w*_) in producing a torque around the shoulder joint. Because the lever arm of the pectoralis is much shorter than this, it needs to act with a force of 7*mg*, where *m* is mass and *g* is gravitational acceleration. The maximum force that can be sustained by the pectoralis is roughly 3 to 4 times the maximum load that the muscle is required to take in gliding flight [39]. With the wings fully outstretched, this would imply a maximum acceleration of 3*g* to 4*g*, corresponding to an aerodynamic load of 1.5*mg* to 2*mg* on each wing, and hence to an aerodynamic moment of 0.75*mgl*_*w*_ to 1*mgl*_*w*_. Pennycuick and Tucker propose the scaling law of a maximum lift of 1.7*mg* for fully stretched wings, which corresponds to a torque force of 0.85*mgl*_*w*_ per wing, exactly in the middle of the measurements by Bayer *et al*. [39]. When a bird retracts its wings, the moment arm decreases. Hence the wing’s maximum load increases. Tucker’s model reveals that peregrine falcons, with appropriately retracted wings, may experience forces of over 18mg during a pull-out of the stoop, resulting in 9mg per wing.

Aerodynamic loads also place pressure on the ligaments and bones. Pennycuick [49] extensively examined the strength of pigeons’ (*Columba livia*) wing bones. He investigated the breaking points in bending and twisting of the humeri and the radio-ulna. Under simple assumptions of the lift distribution on the wing during gliding flight and with fully stretched wings, the lift would need to exceed 16.8 N before torsional or bending pressures would break the bones. As the weight of his pigeons was on average 0.393 kg, this would imply that the wings could hold up to 8 or 9 times the body weight, roughly equivalent to the maximum load that the falcon’s pectoralis can hold when it stoops at high speed. Therefore, the constraint on torque implicitly contains the constraint on aerodynamic loads due to breaking of bones. Further evidence that the peregrine falcon is well adapted to cope with the large aerodynamic forces that arise during maneuvers in a high-speed stoop is provided by Schmitz *et al*. [50]. These authors investigated the mechanical properties of the feathers of four species, including the peregrine falcon, and found that the feathers of the falcon had larger cross-sections and protrusions than the other species, resulting in a greater bending stiffness, thereby allowing greater aerodynamic loads.

#### H Justification of the inertia equation in the model

Here, we derive Eq 32 that determines the whole body inertia in the model for a given wing retraction. The total roll moment of inertia *I*_*x*′_ is the sum of the inertia of the body *I*_*b*_ and the inertia of the wings *I*_*wing*_.

*H.1 Body moment of inertia.* The body inertia about the roll axis is only available for one species (the rose breasted cockatoo; Table 2 of [41]). We apply several allometric scaling laws to determine the inertia of other species based on these data. According to Nudds and Rayner [42], we can adopt the body frontal area and mass relation:

$$\begin{array}{c} S_{b}=0.0066m_{b}^{0.68} \end{array}$$ (80)

The scaling of the body width has the relation

$$\begin{array}{c} w_{b}=0.098m_{b}^{0.35} \end{array}$$ (81)

Because these approximately preserve relationships in scaling, the inertia of the body in the roll direction scales as follows:

$$\begin{array}{c} I_{b}=I_{b0}w_{b}^{2}m_{b}=I_{b0}0.098^{2}m_{b}^{1.70} \end{array}$$ (82)

Where *I*_*b*0_ is the inertia for a width of 1m and a mass of 1kg. The rose breasted cockatoo weighs 0.293 kg with *I*_*b*_ = 1685.5 ⋅ 10^−7^ kg m^2^. Therefore, we estimate *I*_*b*0_ to be 0.1346717. With *I*_*b*0_, we can now estimate the body inertia for other birds. For instance, using these equations, the *I*_*b*_ of a male Peregrine falcon is estimated to be 448.0 ⋅ 10^−6^ kg m^2^.

*H.2 Wing moment of inertia.* The wing inertia has been calculated for many species by Rayner and Van Den Berg [43]. Their calculations determine the moment of inertia about the attachment site of the wing to the body (i.e. the shoulder joint), whereas we are interested in calculating the moment of inertia of the wings about the center of mass of the bird. There can, of course, be a considerable difference between these two quantities (see Table 2 of [41]), but they are easily inter-converted as follows. The moment of inertia about the center of mass is given by:

$$\begin{array}{c} I_{wing.center}=\sum_{i=1}^{n}m_{i}{\left(r_{i}+x\right)}^{2}=\sum_{i=1}^{n}m_{i}r_{i}^{2}+\sum_{i=1}^{n}m_{i}x^{2}+\sum_{i=1}^{n}m_{i}2r_{i}x \end{array}$$ (83)

where *m*_*i*_ is the mass of the *i*th blade element of the wing, *r*_*i*_ its distance from the shoulder joint, and *x* the distance from the shoulder joint to the center of gravity. We may restate this equation in terms of the total mass of the wing *m*_*wing*_ and its total moment of inertia about the shoulder joint *I*_*wing*_ as follows:

$$\begin{array}{c} I_{wing.center}=I_{wing}+x^{2}m_{wing}+2x\sum_{i=1}^{n}m_{i}r_{i} \end{array}$$ (84)

where the summation may be calculated using data from Rayner & Van den Berg [43] and Hedrick & Biewener [30, 41] regarding the mass distribution of the wing. We assume that the distance *x* from the shoulder to the center of mass is half the body width. Empirically, if we do not know the wing length, but do know the body mass, then we can approximate the wing inertia as follows:

$$\begin{array}{c} I_{wing.center}=I_{wing}+\frac{1}{4}0.098^{2}m_{b}^{0.70}m_{wing}+0.098m_{b}^{0.35}J \end{array}$$ (85)

where *J* is the summation term of Eq 32. And, with a decreased wing span, we calculate:

$$\begin{array}{c} I_{wing.center}=I_{wing}\phi^{2}+\frac{1}{4}0.098^{2}m_{b}^{0.70}m_{wing}+0.098m_{b}^{0.35}J\phi \end{array}$$ (86)

where *l*_*wing*_ is the wing length and where *ϕ* is the fraction of wing retraction defined as:

$$\begin{array}{c} \phi =\frac{l_{wing}}{l_{wing.max}} \end{array}$$ (87)

Thus the total roll moment of inertia at a given point in time (assuming wing retraction is symmetrical such that the center of gravity does not change) is:

$$\begin{array}{c} I_{x^{\prime }}=I_{b}+2\left(I_{wing}\phi^{2}+\frac{1}{4}0.098^{2}m_{b}^{0.70}m_{wing}+0.098m_{b}^{0.35}J\phi \right) \end{array}$$ (88)

#### I Proof that the wing span that maximizes lift also maximizes roll acceleration

Here, we explain why Eq 19 maximizes the roll acceleration with respect to wing span. Let us call this maximizing wing span b*. Roll acceleration is maximized when one wing produces the largest positive lift, and the other wing the largest negative lift. The roll acceleration depends on the wing span in the following way:

$$\begin{array}{c} \dot{\omega }=\frac{M}{I}=\frac{\frac{b_{max}}{4}\phi^{2}S_{max}c_{l}\rho v^{2}}{I_{b}+2\left(I_{wing}\phi^{2}+c+0.098m_{b}^{0.35}J\phi \right)} \end{array}$$ (89)

with the constraint that *M* ≤ *M*_*max*_. When *b* > b* the torque will not change, but the inertia will increase, so the roll acceleration is less. When *b* < b*, the torque declines by *ϕ*^2^, while the inertia declines less than *ϕ*^2^. Therefore, b* maximizes the roll acceleration.

#### J Derivation of optimal wing retraction

The object of optimizing wing retraction is to maximize the net thrust minus drag, given a desired lift L*. We assume a simple relation between wing retraction, aspect ratio and wing area, as proposed by Tucker [11]:

$$\begin{array}{c} S=S_{max}\frac{b-b_{min}}{b_{max}-b_{min}} \end{array}$$ (90)

$$\begin{array}{c} A\;R=\frac{b^{2}}{S}=\frac{b^{2}}{S_{max}\frac{b-b_{min}}{b_{max}-b_{min}}} \end{array}$$ (91)

Assuming this relation, the total induced drag becomes:

$$\begin{array}{c} D_{i}=\frac{c_{l}^{2}}{\pi A\;R}\rho \frac{1}{2}Sv^{2}=\frac{S_{max}^{2}{\left(\frac{b-b_{min}}{b_{max}-b_{min}}\right)}^{2}}{2b^{2}}c_{l}^{2}\rho v^{2} \end{array}$$ (92)

and the total thrust minus drag is

$$\begin{array}{c} \text{T}\;D=-\frac{S_{max}^{2}{\left(\frac{b-b_{min}}{b_{max}-b_{min}}\right)}^{2}}{2b^{2}}c_{l}^{2}\rho v^{2}-c_{d.fric}S_{max}\frac{b-b_{min}}{b_{max}-b_{min}}\frac{1}{2}\rho v^{2}-D_{body}+mg\text{cos}\left(\beta \right) \end{array}$$ (93)

where *β* is the dive angle (i.e. the negative of the elevation angle). The optimization becomes:

$$\begin{array}{c} \max_{cl,b}\left\{\text{T}\;D\right\} \end{array}$$ (94)

with the equality constraint that lift equals some desired value:

$$\begin{array}{c} L*=\frac{1}{2}c_{l}\rho S_{max}\frac{b-b_{min}}{b_{max}-b_{min}}v^{2} \end{array}$$ (95)

and the inequality constraints:

$$\begin{array}{c} -c_{l.max}<c_{l}<c_{l.max},b_{min}<b<b_{max} \end{array}$$ (96)

The optimization problem turns out to have a simple solution. Namely, we calculate the partial derivative with respect to *b*

$$\begin{array}{c} \frac{\partial }{\partial b}(-\frac{2{\left(L^{*}\right)}^{2}}{b^{2}\rho v^{2}}-c_{d.fric}S_{max}\frac{b}{b_{max}-b_{min}}\frac{1}{2}\rho v^{2}+mg\;\text{cos}\left(\beta \right)-D_{body})\\ \;+\frac{\partial }{\partial b}(c_{d.fric}S_{max}\frac{b_{min}}{b_{max}-b_{min}}\frac{1}{2}\rho v^{2}) \end{array}$$ (97)

then set it to zero

$$\begin{array}{c} -\frac{4{\left(L^{*}\right)}^{2}}{-2\pi b^{3}\rho v^{2}}-\frac{c_{d.fric}S_{max}}{2(b_{max}-b_{min})}\rho v^{2}=0 \end{array}$$ (98)

and solve for *b*:

$$\begin{array}{c} b=\sqrt[{3}]{\frac{\left(b_{max}-b_{min}\right)8{\left(L^{*}\right)}^{2}}{c_{d.fric}S_{max}{\left(\rho v^{2}\right)}^{2}}} \end{array}$$ (99)

When *b* is less than *b*_*min*_, b is set to *b*_*min*_. This new value of *b* may cause *c*_*l*_ to exceed the constraint. Therefore, we set *c*_*l*_ to *c*_*l*.*max*_, and solve for the corresponding *b*. If *b* is greater than *b*_*max*_, we set it to *b*_*max*_ and solve for *c*_*l*_.

#### K Comparison between flight performance in the model and empirical measurements

In order for the model results to be relevant for our understanding of real falcons, the flight performance of model-birds should approximate empirical measurements in falcons and starlings, as flight performance is expected to be a key determinant of catch success in a chase. However, very little is known about the peak flight performance of birds. Most of the flight performance mentioned elsewhere is derived using modeling, and we have mentioned how our modeling relates to previous models throughout the section A-J. Here, we present the few coarse comparisons that are currently possible. First, minimum sustained flight speeds (the minimum speed of the model-starling is 4.5ms^−1^ and that of the falcon 7.3ms^−1^; defined as the minimum airspeed where acceleration > 0 in Fig 3a) correspond closely with the minimum speeds at which birds fly in wind tunnel experiments (starlings are not reported to fly below 6ms^−2^ [52, 55, 56]). Warrick [57] reports that the linear acceleration of a starling is approximately 5.8ms^−2^ when released to fly through a 2x2x5 tunnel, and our model-starlings linearly accelerate 4.8ms^−2^. The top horizontal speed of peregrine falcons has been found to be 27.6ms^−1^ [58], which is very close to the speed of 28.4ms^−1^ in the model. Starlings have been reported to occasionally fly at 22ms^−1^ during migration [59], so the top speed of 24ms^−1^ in the model also seems appropriate. The maximum dive speed of the falcon is 122ms^−1^ and resembles the speed of 108ms^−1^ achieved by a peregrine falcon in an unpublished study by National Geographic (to date, no peer-reviewed articles include measures of the peak performance of falcons in a stoop). The maximum load factors attained in the model are considerably higher than those measured experimentally. Ponitz *et al*. [3] report that the load factor of their peregrine falcon during pull out was 1.15 (including gravity) at a speed of 20–22ms^−1^. Model-falcons are able to generate a load factor of 2.5 at that speed (excluding gravity), but it is reasonable to assume that a real falcon will generally pull out with a load factor below maximum, to maintain stability throughout its flight. Lastly, the high roll accelerations of our model birds are of the same order of magnitude as the roll accelerations measured in pigeons (*columba livia*) [60]: pigeons had an average whole body angular acceleration of 601 rad s^−2^ flying 3–6 ms^−1^. At that speed, a model-starling exerts ∼1200 rad s^−2^ and a model-falcon ∼400 rad s^−2^.

#### L Tests of convergence of numerical integration

In our simulations, we discretized time and constructed a set of ordinary difference equations to numerically solve the initial value problem of the original differential equations (see Eqs 2, 3, 4, 6 and 7). Here we test the convergence of the position, speed and acceleration of predator and prey, as well as the falcon’s catch success. As we do not have access to the algebraic solutions, we tested the convergence as follows. The time step of integration in the simulations was reduced iteratively in orders of magnitude until an acceptable discrepancy with next step size of integration was obtained, where we define the discrepancy as:

$$\begin{array}{c} e_{dt}=\frac{1}{k}\sum_{n=1}^{k}\left\|f{\left(dt/10\right)}^{10n}-f{\left(dt\right)}^{n}\right\| \end{array}$$ (100)

where *e*_*dt*_ is the discrepancy, *k* the number of model time steps, *f*(*dt*) the difference equation describing the value of interest, where *dt* is the applied time step. We simulated trajectories of stooping falcons (800m altitude and 600m horizontal distance) against erratically or circularly moving prey, where the initial seeding of random variables was the same for each applied time step *dt* and *k* was chosen such that the falcon intercepted the starling in simulations using one of the time step sizes, and thus for no *dt* sizes the simulation continued beyond interception. An acceptable discrepancy was achieved for *dt* = 1 ⋅ 10^−4^ *s* (see Table 5 and S4 Fig). Between *dt* = 1 ⋅ 10^−4^ *s* and *dt* = 1 ⋅ 10^−5^ *s* the catch success varied only 0.13% which is much lower than the minimum difference in main results between tested conditions (2%). Beyond *dt* = 1 ⋅ 10^−6^ *s*, *e*_*dt*_ increases due to floating point imprecision.

**Table 5:** Test of convergence. Falcons stoop from 800m altitude and 600m horizontal distance from the prey. The prey flies erratically. See Eq 100 for a definition of *e*_*dt*_.

measure	*e*_10^−1^_	*e*_10^−2^_	*e*_10^−3^_	*e*_10^−4^_	*e*_10^−5^_
distance predator to prey (m)	246.4	11.2	1.1	0.12	4.3
speed predator (ms^−1^)	23.1	1.1	0.1	0.03	0.9
acceleration predator (ms^−2^)	53.2	8.1	0.3	0.02	20.2
speed prey (ms^−1^)	12.7	2.6	0.4	0.02	1.0
acceleration prey (ms^−2^)	22.8	1.9	0.2	0.03	1.0
catch success (%)	63.2	1.2	0.22	0.13	0.14

Additionally, we have tested the convergence and stability of the numerical integration of Eq 6, in a scenario where convergence is expected to be weak on theoretical grounds. Namely, when the birds exert large roll accelerations, oscillations around the equilibrium bank angle may arise at larger *dt*. S5 Fig shows the bank angle change over time of a falcon stooping at 100ms^−1^, and exerting a roll acceleration of 5000 rad s^−2^ using Bang-Bang control to turn *pi* rad. The maximum absolute error of a *dt* = 1 ⋅ 10^−4^ *s* with respect to *dt* = 1 ⋅ 10^−5^ *s* is only 0.007 rad, which is unlikely to affect the model outcome. We note that, although the model equations are nonlinear, they are inherently stabilizing (i.e. robust to perturbations due to the numerical scheme), as aerodynamic drag ensures that the speed converges towards an equilibrium point, and the guidance law ensures that the falcon is attracted to the prey (see the smooth variation of catch success with respect to variation in initial conditions in Figs 4 and 6).

## Supporting information

S1 Fig Prey accelerations. The figure portrays the distribution of prey accelerations along the x, y and z axes of the inertial axis system, for smooth and non-smooth maneuvering prey.
(EPS)

S2 Fig Flight trajectories. Examples of flight trajectories of the falcon when hunting non-smooth maneuvering prey, and when the navigation constant *N* of the falcon is low (*N* < 2). In some curved flight paths, the peregrine falcon drops below the prey; a phenomenon also observed in nature.
(EPS)

S3 Fig Alternative delay implementation. In our model, a parameter *τ* affects both the differencing time of the line-of-sight, as well as the sample rate from vision to guidance. Here, we show that our results are not implementation specific. When we implement a continuous delay function with analytic differentiation, the same qualitative patterns are found: (a) a peak success at an *N* value of around 3–4, (b) and an increase in success when the falcon dives from a high-altitude.
(EPS)

S4 Fig Example of a trajectory using a time step size 10^−4^s or 10^−5^s. The differences of the trajectories of the prey and predator between step sizes is marginal (i.e. the trajectories converge), and we thus use the larger step size such that run-time of the simulations is minimized.
(EPS)

S5 Fig Change in bank angle over time for different step sizes. It portrays the bank angle change over time of a falcon stooping at 100ms^−1^, and exerting a roll acceleration of 5000 rad s^−2^ using Bang-Bang control to turn π rad.
(EPS)

S1 Video Video of simulated attacks by peregrine falcons in level flight on starlings in smooth maneuvering flight. The camera is set up to follow the falcon from below at a fixed position, mimicking how a human observer would view the spectacle. The falcon misses the starling three times and catches it once. A catch is defined as occurring when the model-falcon comes within 0.2m of the model-starling. A near-miss occurs (“MISS!” appears on the screen in the video) when the model-falcon comes within 5.0 m of the model-starling, but subsequently finds itself further than this from the model-starling and with the model-starling in the blind zone of the model-falcon (a cone of 45° behind the bird).
(AVI)

S2 Video Video of simulated attacks by peregrine falcons in level flight on starlings in straight flight. The starling is caught by the falcon in the two attacks of the video, where a catch occurs when the falcon comes within 20cm of the starling. Colored ribbons behind the birds show their recent trajectories, and follow the motion of the wings; the white marker at fixed time intervals of 0.5s is used to show the speed of the birds along their trajectories. The wingbeat averaged aerodynamic forces due to flapping are analytically derived and act at every time step of the model; there is no explicit model of the tail dynamics. We assume that the lift contribution of the tail is negligible, and that it contributes to the overall body drag measured empirically.
(AVI)

S3 Video Video of simulated attacks by peregrine falcons in level flight on starlings in smooth maneuvering flight. The falcon misses in the first two attacks and catches the starling in the third. A catch is defined as occurring when the model-falcon comes within 0.2m of the model-starling. A near-miss occurs (“MISS!” appears on the screen in the video) when the model-falcon comes within 5.0 m of the model-starling, but subsequently finds itself further than this from the model-starling and with the model-starling in the blind zone of the model-falcon (a cone of 45° behind the bird). Colored ribbons behind the birds show their recent trajectories, and follow the motion of the wings; the white marker at fixed time intervals of 0.5s is used to show the speed of the birds along their trajectories. The wingbeat averaged aerodynamic forces due to flapping are analytically derived and act at every time step of the model; there is no explicit model of the tail dynamics. We assume that the lift contribution of the tail is negligible, and that it contributes to the overall body drag measured empirically.
(AVI)

S4 Video Video of simulated attacks by peregrine falcons in level flight on starlings in non-smooth maneuvering flight. The falcon misses the starling three times and catches it once. A catch is defined as occurring when the model-falcon comes within 0.2m of the model-starling. A near-miss occurs (“MISS!” appears on the screen in the video) when the model-falcon comes within 5.0 m of the model-starling, but subsequently finds itself further than this from the model-starling and with the model-starling in the blind zone of the model-falcon (a cone of 45° behind the bird). Colored ribbons behind the birds show their recent trajectories, and follow the motion of the wings; the white marker at fixed time intervals of 0.5s is used to show the speed of the birds along their trajectories. The wingbeat averaged aerodynamic forces due to flapping are analytically derived and act at every time step of the model; there is no explicit model of the tail dynamics. We assume that the lift contribution of the tail is negligible, and that it contributes to the overall body drag measured empirically.
(AVI)

S5 Video Video of simulated attacks by peregrine falcons in stooping flight on starlings in non-smooth maneuvering flight. The falcon catches the starling three times and misses it once. A catch is defined as occurring when the model-falcon comes within 0.2m of the model-starling. A near-miss occurs (“MISS!” appears on the screen in the video) when the model-falcon comes within 5.0 m of the model-starling, but subsequently finds itself further than this from the model-starling and with the model-starling in the blind zone of the model-falcon (a cone of 45° behind the bird). Colored ribbons behind the birds show their recent trajectories, and follow the motion of the wings; the white marker at fixed time intervals of 0.5s is used to show the speed of the birds along their trajectories. The wingbeat averaged aerodynamic forces due to flapping are analytically derived and act at every time step of the model; there is no explicit model of the tail dynamics. We assume that the lift contribution of the tail is negligible, and that it contributes to the overall body drag measured empirically.
(AVI)
